# Regulatory networks of FUR and NtcA are intertwined by transcriptional regulators, two-component systems, serine/threonine kinases, and sigma factors in *Anabaena* sp. PCC 7120

**DOI:** 10.1128/msystems.00373-25

**Published:** 2025-06-25

**Authors:** J. Guío, M. L. Peleato, M. F. Fillat, E. Sevilla

**Affiliations:** 1 Department of Biochemistry & Molecular and Cellular Biology, University of Zaragoza 16765, Zaragoza, Spain; 2 Institute for Biocomputation and Physics of Complex Systems (BIFI), Zaragoza, Spain; UiT Norges arktiske universitet Arctic Centre for Sustainable Energy, Tromsø, Norway

**Keywords:** regulatory networks, *Anabaena *sp. PCC 7120, FUR (ferric uptake regulator), NtcA, transcriptional regulator, two-component system, serine/threonine kinase, sigma factor

## Abstract

FUR proteins in *Anabaena* sp. PCC 7120 (FurA/Fur, FurB/Zur, and FurC/PerR) are a family of transcriptional regulators involved in the control of highly important metabolic processes such as the maintenance of metal homeostasis, the regulation of oxidative stress response, and the adaptation to nitrogen starvation. Previous RNAseq analyses of FUR misregulation strains revealed a broad panel of genes directly modulated by these transcriptional regulators. However, the expression of several regulatory proteins was also altered, indicating that FUR proteins could extend their influence by exerting a second level of regulation through some members of their regulons. In this work, by combining differential gene expression data and electrophoretic mobility shift assays (EMSAs), we sought to identify novel direct targets of FUR proteins with regulatory functions, namely, transcriptional regulators, two-component systems, sigma factors, and other proteins with regulatory functions such as serine/threonine kinases. This allowed us to build a network composed of these regulatory proteins that are directly modulated by FUR proteins. In addition, taking into account the role of FUR proteins in the regulation of nitrogen metabolism, the overlap between FUR and NtcA regulatory networks was studied, revealing that an important part of the FUR network is coregulated by NtcA. These results unveil a complex network in *Anabaena* in which regulatory proteins hierarchically below FUR or NtcA proteins could be controlling the expression of several genes, connecting the integration of stress signaling performed by FUR and NtcA to a wide set of cyanobacterial transcriptional responses.

**IMPORTANCE:**

FUR proteins in *Anabaena* sp. PCC 7120 are a family of global transcriptional regulators that control several cellular processes ranging from metal homeostasis to nitrogen metabolism. Apart from directly regulating their target genes, the differential expression of several regulatory genes in RNAseq analyses of FUR misregulation strains suggests that these transcriptional regulators could also control the expression of many targets indirectly. Here, we report that FUR proteins from *Anabaena* sp. PCC 7120 directly modulate the expression of transcriptional regulators, two-component systems, sigma factors, and serine/threonine kinases, revealing that these regulators indirectly modulate a wide number of genes and cellular processes through this regulatory network. Besides, it was found that an important part of this network is co-regulated by NtcA, connecting the integration of FUR and NtcA stress signals and suggesting that the FUR regulatory network could be involved in the adaptive responses to nitrogen deficiency.

## INTRODUCTION

Cyanobacteria can live in a wide variety of environments, which are characterized by continual fluctuations in physicochemical parameters like salinity, oxygen tension, or nutrient availability. For this reason, they have developed mechanisms to face and adapt to these stressful situations. Stress signaling in cyanobacteria is mainly modulated by gene expression control through transcriptional regulators, two-component systems, sigma factors, and antisense RNAs (1, 2). Transcriptional regulators are proteins capable of binding to specific promoter regions of their target genes, thereby activating or repressing their expression. These regulators usually hold a sensor domain and a helix-turn-helix DNA-binding domain, and they can modulate gene expression in response to stress signals (3). The genome of *Anabaena* sp. PCC 7120 (also known as *Nostoc* sp. PCC 7120) contains approximately 100 genes annotated as transcriptional regulators. Similarly, but using a more complex mechanism, two-component systems coordinate the response to stress situations. Two-component systems are composed of a histidine kinase and a response regulator. Histidine kinase, also known as sensor kinase, holds the sensor domain which, in the presence of the signal, promotes its activation by autophosphorylation. Then, the phosphate is transferred to its cognate response regulator, activating it (4). Response regulators usually act as transcriptional regulators, but they sometimes display enzymatic activities or bind to RNAs or other proteins (4, 5). In *Anabaena,* 211 gene sequences were identified as two-component systems signaling elements, but most of them have unknown functions (6). Sigma factors are dissociable subunits of RNA polymerase which can modulate its efficiency and specificity (7). When the sigma subunit binds to the core of RNA polymerase, generally in response to a certain stress, the complete holoenzyme is able to bind to specific promoters and initiate their transcription (7). In this way, bacteria are able to regulate gene expression to face different stress situations (7). In bacteria, sigma factors are divided into two major families, σ^54^ and σ^70^ (8). In cyanobacteria, homologs of the σ^54^ family have not been found, but several homologs of the σ^70^ family have been identified. The σ^70^ family is divided into four groups. In particular, *Anabaena* sp. PCC 7120 contains 12 sigma factors belonging to the σ^70^ family.

Group 1 is constituted by *sigA* (*all5263*), which recognizes promoters of constitutive essential genes, whereas group 2 is formed by *sigB2* (*alr3800*), that regulates the response to saline stress (9), and *sigC* (*all1692*), *sigD* (*alr3810*), and *sigE* (*alr4249*), that are reported to be involved in proheterocyst differentiation and heterocyst development (10, 11). In addition, paralogs of *sigB2*, named *sigB*, *sigB3,* and *sigB4,* which are located in plasmids, also belong to this group. Group 3 is formed by *sigF* (*all3853*) and *sigJ* (*alr0277*), being the latter involved in the tolerance to desiccation and photoprotection (11–13). Finally, *sigG* (*alr3820*), which seems to be involved in the mechanism of commitment of heterocyst differentiation, and *sigI* (*all2193*), whose role is unknown, constitute group 4 (11).

The three proteins that make up the FUR family in *Anabaena* sp. PCC 7120, called FurA, FurB (Zur), and FurC (PerR), and the NtcA protein function as master regulators in this cyanobacterium (14–20). Broadly speaking, FUR proteins are metalloregulators mainly involved in the maintenance of metal homeostasis and redox state of the cell (21, 22). More specifically, FurA is known as the master regulator of iron homeostasis, and FurB (Zur) controls fundamentally zinc uptake and transport, whereas FurC (PerR) is mainly responsible for the response of *Anabaena* to oxidative stress (21, 22). However, in the last years, transcriptomic analysis of FUR misregulation strains vs the wild-type *Anabaena* sp. PCC 7120 revealed that these regulators are engaged in the regulation of other important processes such as carbon metabolism, photosynthesis, biofilm formation, or the adaptation to nitrogen deficiency (14, 16–18). Concerning NtcA, this protein is a master regulator belonging to the CRP (cAMP receptor protein) family that controls the responses of the cell to nitrogen availability (23). Upon nitrogen starvation, an increase in 2-oxoglutarate (2-OG) levels activates NtcA, which is present at low levels under nitrogen-replete conditions. Then, NtcA binds to its own promoter, increasing the levels of the NtcA protein and, in turn, triggering the transcriptional response of this global regulator (24). Most promoters of genes that are activated by NtcA comprise the NtcA consensus binding site (GTAN_8_TAC) (25). Chromatin immunoprecipitation followed by high-throughput sequencing (ChIP-seq) allowed the identification of NtcA-binding sites in the *Anabaena* sp. PCC7120 genome. These analyses revealed that NtcA regulates the expression of genes belonging to nitrogen metabolism but also genes involved in iron and carbon metabolism or photosynthesis (20).

Both FUR and NtcA are master regulators that directly control the expression of multiple genes. They function as transcriptional regulators by binding to the promoters of genes involved in various stress responses, modulating their transcription by either repressing or activating their expression. However, many other genes that change their expression profile in their misregulation strains are indirectly regulated by them. Interestingly, when the transcriptomes of FUR variants were analyzed, alterations in the expression of several genes with regulatory functions were found. In the transcriptome of a conditional deletion mutant of *furA* (*coaR*-P*_coaT_::furA* fusion strain, AGcoaRFurA)*,* 175 genes with regulatory functions showed altered expression (14). Similarly, the transcription of 14 genes or transcriptional units with regulatory functions was affected in a *furB*-deletion strain (Δzur) (16), and 36 genes changed their expression in a *furC*-overexpressing strain (EB2770FurC) (17). Additionally, ChIP-seq analysis performed with NtcA in *Anabaena* sp. PCC7120 revealed that 179 genes with regulatory functions were potentially controlled by NtcA (20).

In the present work, we investigated the indirect regulation mediated by FurA, FurB, and FurC proteins by identifying their direct targets among various regulatory proteins, including transcriptional regulators, two-component systems, and sigma factors whose expression is altered in FUR misregulation strains. Additionally, we analyzed regulatory proteins directly controlled by FUR as potential direct targets of NtcA. This information allowed us to outline a scheme of the global regulatory network governed by FUR proteins and establish its interconnection with the NtcA regulatory network.

## RESULTS

### Regulatory network controlled by FurA

Previous transcriptional studies of the conditional deletion mutant of *furA* (AGcoaRFurA) (14) revealed that the expression of 175 genes involved in regulatory functions was altered in this strain (14). Among them, we selected those genes with predicted FurA-boxes in their promoter region (15) and also regulatory genes with known functions, namely, *calA*, *calB*, *rpaA*, *cyaB2*, *sixA*, *pkn22*, *prpA*, *pknE,* and *pknD*. These genes are listed in Table 1, indicating the fold change found in the differential transcriptomic analysis of the conditional deletion mutant of *furA* (AGcoaRFurA). The binding of FurA to the promoter region of some regulatory genes had already been tested in other studies carried out by our group, yielding positive results (15, 26). This is indicated in Table 1 as [+], including the reference in which those assays can be found.

**TABLE 1:** Genes with regulatory functions containing a FurA DNA-binding box (15) and showing differential expression in the conditional deletion mutant of *furA* (14)^*a*^

Putative transcriptional unit^*b*^	Gene name/protein description	Fold change	EMSA FurA^*c*^
Transcriptional regulators			
*all1651*	*all1651*; transcriptional regulator	−3.4	**+**
*all2080*	***calB***; AbrB family transcriptional regulator	−2.9	**+**
*all3903*	*all3903*; ArsR family transcriptional regulator	1.5	**+**
*alr0946*	***calA***; AbrB family transcriptional regulator	−3.1	**–**
Two-component systems			
*all0129*	***rpaA***; response regulator	−2.0	**+**
*all1704*	*all1704*; response regulator	1.9	**–**
*all1804*	*all1804*; hybrid histidine kinase	−2.1	**+**
*all2699*	***aphC***; two-component sensor histidine kinase	−14.3^*f*^	**[+]^*d*^**
*all7584-83*	*all7583*; histidine kinase	−1.9	**+**
	*all7584*; response regulator		
*alr0072*	*alr0072*; two-component response regulator	−1.6	**+**
Serine/threonine kinases			
*alr2411-12*	*alr2411*; serine/threonine kinase	−6.6	**–**
	*alr2412*; serine/threonine kinase	−3.5	
*alr2502*	***pkn22***; serine/threonine kinase	−3.4	**[+]^*e*^**
*alr3732*	***pknE***; serine/threonine kinase	−2.4	**+**
*alr4368*	***pknD***; serine/threonine kinase	−2.3	**+**
Other genes with regulatory functions			
*all0743*	***cyaD***; adenylate cyclase	−11.1^*f*^	**[+]^*d*^**
*all1904*	***cyaB2***; adenylate cyclase	−2.1	**+**
*all2416*	*all2416*; putative diguanylate cyclase	−2.5	**–**
*all4379*	*all4379*; peptide-chain-release factor 3	−2.1	**+**
*all4896*	*all4896*; putative diguanylate cyclase with PAS/PAC sensor	−5.1	**+**
*all4963*	***cyaC***; adenylate cyclase carrying two-component sensor and regulator domains	−4.3^*f*^	**[+]^*d*^**
*alr0221*	***sixA***; phosphohistidine phosphatase	−2.8	**+**
*alr3165-66*	***asr***; bacteriorhodopsin	−50.0^*f*^	**[+]^*d*^**
	***asrt***; sensory rhodopsin transducer		
*alr3731*	***prpA***; serine/threonine phosphatase	−3.2	**–**

^*a*^ The specific names of some of the genes that appear in databases or bibliography are highlighted in bold.

^*b*^ Operons were annotated based on data from MicrobesOnline (http://www.microbesonline.org/) and CyanoCyc (https://cyanocyc.org/).

^*c*^ Genes tested by EMSA with FurA showing the result as +/−. The symbols in brackets indicate genes already tested in previous works.

^*d*^ Genes in which the promoter region was tested by EMSA with FurA by González et al. (15).

^*e*^ Genes in which the promoter region was tested by EMSA with FurA by Yingping et al. (26).

^*f*^ Genes in which the transcriptional change was determined by semi-quantitative RT-PCR by González et al. (15).

Binding of FurA to the promoter region of the remaining selected genes was tested by EMSA, allowing the identification of 13 novel regulatory genes or transcriptional units as potential FurA direct targets. Results of EMSA assays are included in Table 1, and EMSA gels of novel FurA direct targets are shown in Fig. S1. As can be seen, the promoters of three transcriptional regulators named *all1651*, *all3903,* and *calB* showed clear band shift retardation so that they can be considered direct targets of FurA. All1651 belongs to the AraC family of transcriptional regulators, which is involved in the response to stresses, carbon metabolism, and pathogenesis (27). All3903 belongs to the ArsR family of metalloregulators that controls the responses to the presence of heavy metals, among others (28). On the other hand, CalB is a transcriptional regulator that belongs to the clade B of cyanobacterial AbrB regulators (29) and, together with CalA, is somehow involved in the adaptation of *Anabaena* to nitrogen deficiency, although its role remains unclear (30).

Furthermore, five genes forming two-component systems were also found as direct targets of FurA. These genes include one complete two-component system composed by *all7583* (sensor kinase) and *all7584* (response regulator), two response regulators (*alr0072* and *rpaA*), and two hybrid sensor kinases (*alr3442* and *all1804*). Although the role of the two-component system *all7583*/*all7584* is unknown, it has been identified as an homolog of *copS*/*copR* (*sll0790*/*sll0789*) from *Synechocystis*, a two-component system that regulates the uptake and assimilation of cooper (6, 31). Likewise, *all0072* is another putative homolog of *copR* (*sll0789*) (6). RpaA is a master regulator involved in the regulation of the circadian clocks in *Anabaena* sp. PCC 7120 (32). Circadian clocks allow organisms to synchronize light/darkness cycles of the Earth with their own cellular processes, and it has been proposed that RpaA could be transducing the signal of the three Kai proteins to clock-controlled genes (32). In turn, *alr3442* and *all1804* are two hybrid histidine kinases with unknown function. Besides, previous works also identified other two-component system genes as FurA direct targets, namely, *aphC* two-component sensor kinase, which triggers the cAMP signaling induced by far-red light (15, 33), and *pkn22* serine/threonine kinase, which is involved in signaling peroxide stress and nitrogen deprivation (26).

Finally, six genes displaying other regulatory functions were also found as FurA direct targets by EMSA assays. These genes include two serine/threonine kinases (*pknE* and *pknD*), a phosphohistidine phosphatase (*sixA*), an adenylate cyclase (*cyaB2*), the peptide-chain-release factor 3 (*all4379*), and a putative diguanylate cyclase with a PAS/PAC sensor domain (*all4896*). PknD and PknE are serine/threonine kinases that regulate the response of *Anabaena* to nitrogen deficiency and are involved in the heterocyst formation (34, 35). SixA is a phosphatase with an unknown function in *Anabaena,* but its ortholog in *E. coli* is responsible for ArcB dephosphorylation. It is noticeable that ArcB is a histidine kinase that controls the oxidative and fermentative catabolism of *E. coli* (36, 37). CyaB2 is an adenylate cyclase that contains a cAMP binding GAF domain, and interestingly, its activity is inhibited by the presence of sodium (38, 39). Curiously, previous results showed that FurA also regulated directly the expression of the adenylate cyclases *cyaC* and *cyaD* (15) that respond to different environmental factors such as light in the case of CyaC (40), indicating the participation of FurA in the modulation of the cAMP signal transduction cascade.

### Regulatory network controlled by FurB

Regarding FurB, a comparative transcriptomic analysis of the *furB* deletion mutant (Δ*zur*) vs the wild-type strain *Anabaena* sp. PCC 7120 was recently conducted by our group (16). In this study, we identified changes in the expression of 14 genes or transcriptional units with regulatory functions, which are included in Table 2, indicating the fold change observed in the differential transcriptomics profile. It is interesting to note that most of them are proteins annotated as two-component systems. The interaction of the promoters of the transcriptional repressor *smtB* and the *asr/asrt* system with FurB was tested previously by EMSA (16), and results are indicated in Table 2 as [+] or [–]. In the present work, we analyzed by EMSA the binding of FurB to the promoters of the remaining genes in order to identify novel direct targets of FurB (Table 2). The positive EMSA gels are shown in Fig. S2.

**TABLE 2:** Genes with regulatory functions showing differential expression in the Δ*zur* strain (14)^*a*^

Putative transcriptional unit^*b*^	Gene name/protein description	Fold change	EMSA FurB^*c*^
Transcriptional regulators			
*all7621*	***smtB*;** transcriptional repressor (zinc)	3.5	**[+]^*d*^**
*all4986*	***ndhR*** orthologue; low CO_2_ responsive transcriptional regulator	−4.0	**–**
Two-component systems			
*all0926*	*all0926*; hybrid sensor kinase	4.8	**–**
*all3767-64*	*all3764*; hybrid sensor kinase	2.7	**–**
	*all3765*; hybrid sensor kinase	5.6	
	*all3766*; response regulator	4.1	
	*all3767*; histidine kinase	4.5	
*alr3768*	***orrA***; two-component response regulator	3.1	**–**
*all5210*	*all5210*; hybrid sensor kinase	2.7	**+**
*all7583-84*	*all7583*; histidine kinase	−5.3	**+**
	*all7584*; response regulator	−3.2	
*all7605-06*	*all7605*; histidine kinase	−6.9	**+**
	*all7606*; response regulator	−9.4	
*alr0072*	*alr0072*; response regulator	4.5	**+**
*alr0428-29*	*alr0428*; histidine kinase	4.4	**–**
	*alr0429*; response regulator	5.5	
*alr7219*	*alr7219*; response regulator	−2.1	**–^*e*^**
*alr8535*	*alr8535*; response regulator	4.8	**–**
Serine/threonine kinases			
*alr0900*	*alr0900*; serine/threonine kinase with two-component sensor domain	3.1	**+**
Other genes with regulatory functions			
*all7218*	*all7218*; histidine kinase-like ATPase	−2.7	**–^*e*^**
*alr3165-66*	***asr***; bacteriorhodopsin	5.8	**[–]^*d*^**
	***asrt***; sensory rhodopsin transducer	10.8	

^*a*^ The specific names of some of the genes that appear in databases or bibliography are highlighted in bold.

^*b*^ Operons were annotated based on data from MicrobesOnline (http://www.microbesonline.org/) and CyanoCyc (https://cyanocyc.org/).

^*c*^ Genes tested by EMSA with FurB showing the result as +/−. The symbols in brackets indicate genes already tested in previous works.

^*d*^ Genes in which the promoter region was tested by EMSA with FurB by Olivan Muro et al. (16).

^*e*^ alr7219 and all7218 share a 327 bp intergenic region, so EMSA results could be associated to either or both.

Two complete two-component systems (*all7606-05 and all7584-83*), one response regulator (*alr0072*), and one hybrid sensor kinase (*all5210*) were found as direct targets of FurB. As it was said before, *all7584-83* could be the homolog of the *copS/copR* system, and it is noteworthy that *all7606-05* is also a putative homolog of the *copS*/*copR* system. The hybrid sensor kinase *all5210* displays an unknown function, and *alr0072* is a putative homolog of *copR*. In addition, the promoter of *all0900,* a serine/threonine kinase whose function remains unknown, was also positive in EMSA assays.

### Regulatory network controlled by FurC

Previous works performed transcriptional analyses comparing a *furC*-overexpressing strain (EB2770FurC) vs *Anabaena* sp. PCC 7120 wild type in nitrogen replete and nitrogen starvation conditions (17). As it can be seen in Table 3, many of the transcriptional changes were only observed under nitrogen starvation conditions. These analyses revealed that 26 genes or transcriptional units with regulatory functions showed differential expression in the *furC*-overexpressing strain (EB2770FurC) (Table 3). Some of them were previously reported to be direct or indirect targets of FurC (17, 18). This is indicated in Table 3 as [+] or [–], including the reference in which those assays can be found. The binding of FurC to the promoters of the remaining 19 genes or transcriptional units was evaluated by EMSA assays in the present work. EMSA gels of positive results are included in Fig. S3.

**TABLE 3:** Genes with regulatory functions showing differential expression in the *furC* overexpression strain (EB2770FurC) (17)^*a*^

Putative transcriptional unit^*b*^	Gene name/protein description	Fold change	Fold change (-NO_3_)	EMSA FurC^*c*^
Transcriptional regulators				
*all0345*	*all0345*; transcriptional regulator		5.7	**+**
*all1651*	*all1651*; transcriptional regulator		3.5	**+**
*all2035*	*all2035*; transcriptional regulator	3.2	5.5	**–**
*all7016*	*all7016*; transcriptional regulator, heme-binding GAF protein	650.0	650.0	**[+]^*d*^**
*alr1941*	*alr1941*; transcriptional regulator, TetR family		−4.1	**–**
*alr1976*	*alr1976*; transcriptional regulator	−13.0	−15.1	**+**
*alr2325*	***ancrpB***; cAMP receptor protein transcriptional regulator	1.5^*f*^	2.7^*f*^	**[+]^*e*^**
Two-component systems				
*all0926*	*all0926*; hybrid sensor kinase		3.5	**–**
*all1071-68*	***pixL***; hybrid sensor kinase		3.8	**–**
	***pixJ***; methyl-accepting chemotaxis protein		3.2	
	***pixI***; positive phototaxis protein			
	***pixH***; response regulator		3.3	
*all1281*	*all1281*; response regulator		3.5	**–**
*all2165-61*	***pixL***; hybrid sensor histidine kinase		2.8	**[+]^*d*^**
	***pixJ***; methyl-accepting chemotaxis protein		3.0	
	***pixI***; positive phototaxis protein		4.3	
	***pixH***; response regulator, chemotaxis family			
	*all2165*; response regulator, PATAN domain		3.2	
*all3359*	*all3359*; histidine kinase		2.8	**+**
*all3564*	*all3564*; histidine kinase		3.0	**–**
*all3788*	*all3788*; response regulator		4.3	**–**
*all5323*	***rcaC*** homolog; response regulator		3.7	**–**
*alr0264*	*alr0264*; two-component system regulatory protein		4.9	**–**
*alr0774*	*alr0774*; response regulator		7.5	**–**
*alr2137-38*	*alr2137*; NarL family histidine kinase	2.0^*f*^	2.6^*f*^	**[+]^*e*^**
	*alr2138*; response regulator			
*alr2572*	***chk34***; histidine kinase	−3.0		**[–]^*d*^**
*alr8535*	*alr8535*; response regulator	650.0		**[–]^*d*^**
*alr9013*	*alr9013*; response regulator	−48.5	−18.6	**[–]^*d*^**
Serine/threonine kinases				
*all0323*	*all0323*; serine/threonine kinase	650.0		**[–]^*d*^**
*all4687*	*all4687*; serine/threonine kinase with two-component sensor domain		3.0	**–**
*alr0354*	*alr0354*; serine/threonine kinase with two-component sensor domain		−39.4	**+**
*alr0709*	*alr0709*; serine/threonine kinase with two-component sensor domain		3.0	**+**
*alr3732*	***pknE***; serine-threonine kinase		3.5	**+**

^*a*^ The specific names of some of the genes that appear in databases or bibliography are highlighted in bold.

^*b*^ Operons were annotated based on data from MicrobesOnline (http://www.microbesonline.org/) and CyanoCyc (https://cyanocyc.org/).

^*c*^ Genes tested by EMSA with FurC showing the result as +/−. The symbols in brackets indicate genes already tested in previous works.

^*d*^ Genes in which the promoter region was tested by EMSA with FurC by Sarasa-Buisan et al. (17).

^*e*^ Genes in which the promoter region was tested by EMSA with FurC by Sarasa-Buisan et al. (18).

^*f*^ Genes in which the transcriptional change was determined by RT-PCR in this work.

EMSAs showed that three transcriptional regulators (*alr1976*, *all0345,* and *all1651*) are direct targets of FurC. All0345 belongs to the MerR family of transcriptional regulators that respond to heavy metals, oxidative stress, and antibiotics (41), while Alr1976 is part of the XRE family, which controls multiple processes such as virulence factors, stress response, metabolic processes, and biofilm formation (42). All1651 belongs to the AraC family of transcriptional regulators. This family, as it was said before, is involved in the response to stresses, carbon metabolism, and pathogenesis (27). Furthermore, a sensor kinase (*all3359*) and three serine/threonine kinases (*alr0354*, *alr0709*, *pknE*) were also positive in the EMSA assays, suggesting that they are directly controlled by FurC. Regarding their function, All3359 is a sensor kinase with unknown function that holds only a histidine kinase domain and lacks a sensor domain. All0354 and Alr0709 are proteins that contain a serine/threonine kinase domain, a GAF sensor domain and a histidine kinase domain. These proteins would be hybrids of serine/threonine kinases and histidine kinases (6). It is interesting to note the high number of genes related to two-component systems that change their expression in the *furC*-overexpressing strain (EB2770FurC), but EMSA assays yielded negative results, indicating that they must be indirectly regulated.

### Some of the regulatory proteins whose expression is controlled by the FUR family are additionally regulated by NtcA

Our interest was not only the establishment of regulatory networks performed by FUR proteins but also to find interconnections with other remarkable regulatory networks in *Anabaena* sp. PC7120, as it is the case of the NtcA network. As mentioned previously, NtcA is recognized as the master regulator of nitrogen metabolism and the FUR paralogs from *Anabaena* sp. PCC 7120 have been found to be engaged in the regulation of this complex process (16, 17, 43). For this reason, using previously reported NtcA ChIP-seq data (20), we looked for the presence of NtcA binding sites in the promoter region of all the regulatory genes that have been described as being directly regulated by FurA, FurB, or FurC in the present or previous works (Table 4). Sixteen transcriptional units were found to have NtcA-binding sites in their promoter region and, consequently, could be potentially regulated by NtcA. The binding of NtcA to these promoter regions was tested by EMSA assays, and in all cases, NtcA was found to be able to bind to them (Fig. S4). This fact strongly suggests that these 16 genes, which encode the regulatory proteins CyaC, CyaD, CalB, All1804, All7584-83, PknE, RpaA, All4379, CyaB2, Alr2137, and Alr0709*,* are coregulated by FUR proteins and NtcA, together with the serine/threonine kinase Pkn22 which was found to be coregulated by FurA and NtcA in previous works (26).

**TABLE 4:** Analysis of the corregulation by NtcA of regulatory genes identified as direct targets of FUR regulators^*a*^

Putative transcriptional unit^*b*^	Gene name/protein description	FurA^*c*^	FurB^*c*^	FurC^*c*^	NtcA^*c*^
Transcriptional regulators					
*all1651*	*all1651*; transcriptional regulator	**A**		**A**	
*all2080*	***calB***; AbrB family transcriptional regulator	**A**			**A**
*all3903*	*all3903*; ArsR family transcriptional regulator	**R**			
*all7621*	***smtB*;** transcriptional repressor (zinc)		**R**		
*all0345*	*all0345*; transcriptional regulator			**A**	
*all7016*	*all7016*; transcriptional regulator, heme-binding GAF protein			**A**	
*alr1976*	*alr1976*; transcriptional regulator			**R**	
*alr2325*	***ancrpB***; cAMP receptor protein transcriptional regulator			**A**	
Two-component systems					
*all0129*	***rpaA***; response regulator	**A**			**+**
*all1804*	*all1804*; hybrid sensor kinase	**A**			**A**
*all7584-83*	*all7583*; histidine kinase	**A**	**A**		**+**
	*all7584*; response regulator				
*alr0072*	*alr0072*; response regulator	**A**	**R**		
*all2699*	***aphC***; histidine kinase	**A**			
*all5210*	*all5210*; hybrid sensor kinase		**R**		
*all7605-06*	*all7605*; histidine kinase		**A**		
	*all7606*; response regulator				
*alr2137-38*	*alr2137*; NarL family, sensor kinase			**A**	**+**
	*alr2138*; response regulator				
*all3359*	*all3359*; histidine kinase			**A**	
*all2165-61*	***pixL***; hybrid sensor kinase			**A**	
	***pixJ***; methyl-accepting chemotaxis protein				
	***pixI***; positive phototaxis protein				
	***pixH*** response regulator, chemotaxis family				
Serine/threonine kinases					
*alr2502*	***pkn22***; serine/threonine kinase	**R^*d*^**			**A^*d*^**
*alr3732*	***pknE***; serine/threonine kinase	**A**		**A**	**R**
*alr4368*	***pknD***; serine/threonine kinase	**A**			
*alr0900*	*alr0900*; serine/threonine kinase with two-component sensor domain		**R**		
*alr0354*	*alr0354*; serine/threonine kinase with two-component sensor domain			**R**	
*alr0709*	*alr0709*; serine/threonine kinase with two-component sensor domain			**A**	**A**
Other genes with regulatory functions					
*all0743*	***cyaD***; adenylate cyclase	**A**			**A**
*all1904*	***cyaB2***; adenylate cyclase	**A**			**A**
*all4379*	*all4379*; peptide-chain-release factor 3	**A**			**A**
*all4896*	*all4896*; putative diguanylate cyclase with PAS/PAC sensor	**A**			
*all4963*	***cyaC***; adenylate cyclase carrying two-component sensor and regulator	**A**			**+**
*alr3165-66*	***asr***; bacteriorhodopsin	**A**			
	***asrt***; sensory rhodopsin transducer				

^*a*^ The specific names of some of the genes that appear in databases or bibliography are highlighted in bold.

^*b*^ Operons were annotated based on data from MicrobesOnline (http://www.microbesonline.org/) and CyanoCyc (https://cyanocyc.org/).

^*c*^ Direct targets of FUR proteins and NtcA are marked “A” if FUR/NtcA has been proposed to work as activator and “R” if FUR/NtcA has been proposed to work as repressor. In the case of NtcA, genes for which the differential expression in CSE2 strain was under the cutoff (Fig. 1), and thus the effect of NtcA on its expression cannot be determined, are indicated with “+”.

^*d*^ Influence of FurA and NtcA on gene expression according to Yingping et al. (26).

**Fig 1:**
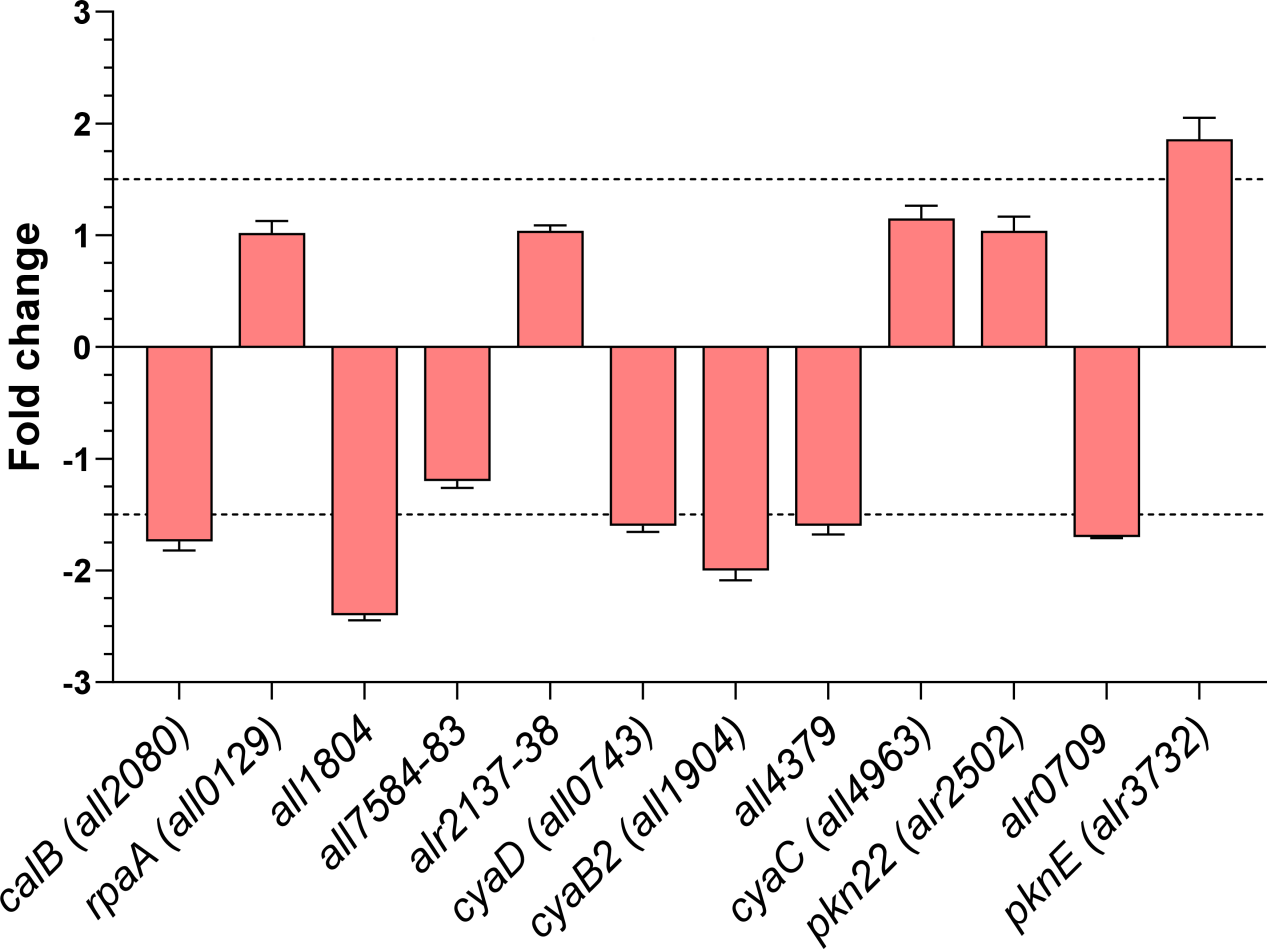
Transcriptional analysis of the novel NtcA direct targets in a *ntcA* deletion strain. Influence of *ntcA* deletion on the mRNA levels of genes with regulatory functions for which direct binding of NtcA to their promoter region was observed by EMSA. Relative real-time RT-PCR was used. Values are expressed as fold change (*ntcA* deletion strain [CSE2] vs wild-type strain) and correspond to the average of three independent assays; the standard deviation is indicated.

Transcriptional analyses were conducted to compare the expression levels of the novel NtcA direct targets in Δ*ntcA* strain vs the wild-type strain *Anabaena* sp. PCC 7120. Most of the tested genes showed altered expression in the CSE2 strain, thus confirming their direct regulation by NtcA (Fig. 1). Interestingly, most of the genes with differential expression were downregulated in the *ntcA* deletion strain, suggesting that NtcA would be acting as an activator of their expression. The sole exception was the gene *pknE*, which was overexpressed.

### FUR family and NtcA bind to the promoter regions of several sigma factors

Apart from transcriptional regulators, two-component systems, and serine/threonine kinases, sigma factors are also important players in the control of gene expression. For this reason, the binding of FUR proteins to the promoter regions of the 12 sigma factors of *Anabaena* sp. PCC7120 was tested in EMSA assays. Positive results are included in Fig. S5. FurA was able to bind to the promoter regions of *sigA*, *sigB*, *sigB3*, *sigB4*, *sigD*, *sigE*, *sigF*, *sigG,* and *sigJ* (Fig. S5A). FurB was able to bind to the promoter regions of *sigA*, *sigB*, *sigB3*, *sigB4*, *sigD*, *sigE,* and *sigJ* (Fig. S5B). Likewise, FurC was able to bind to the promoter regions of *sigA*, *sigD*, *sigB2*, *sigB3,* and *sigI* (Fig. S5C). Also, previous studies found that FurA bound to the promoter region of *sigC* (44).

Finally, in order to examine which of those sigma factors were also regulated by NtcA, the binding of NtcA to their promoter regions was evaluated by EMSA assays. Results shown in Fig. S6 revealed that NtcA binds to the promoter of *sigA*, *sigB*, *sigD*, *sigE*, *sigG*, *sigI*, *sigB3,* and *sigB4*. Also, previous studies found that NtcA bound to the promoter region of *sigC* (44).

**Fig 2:**
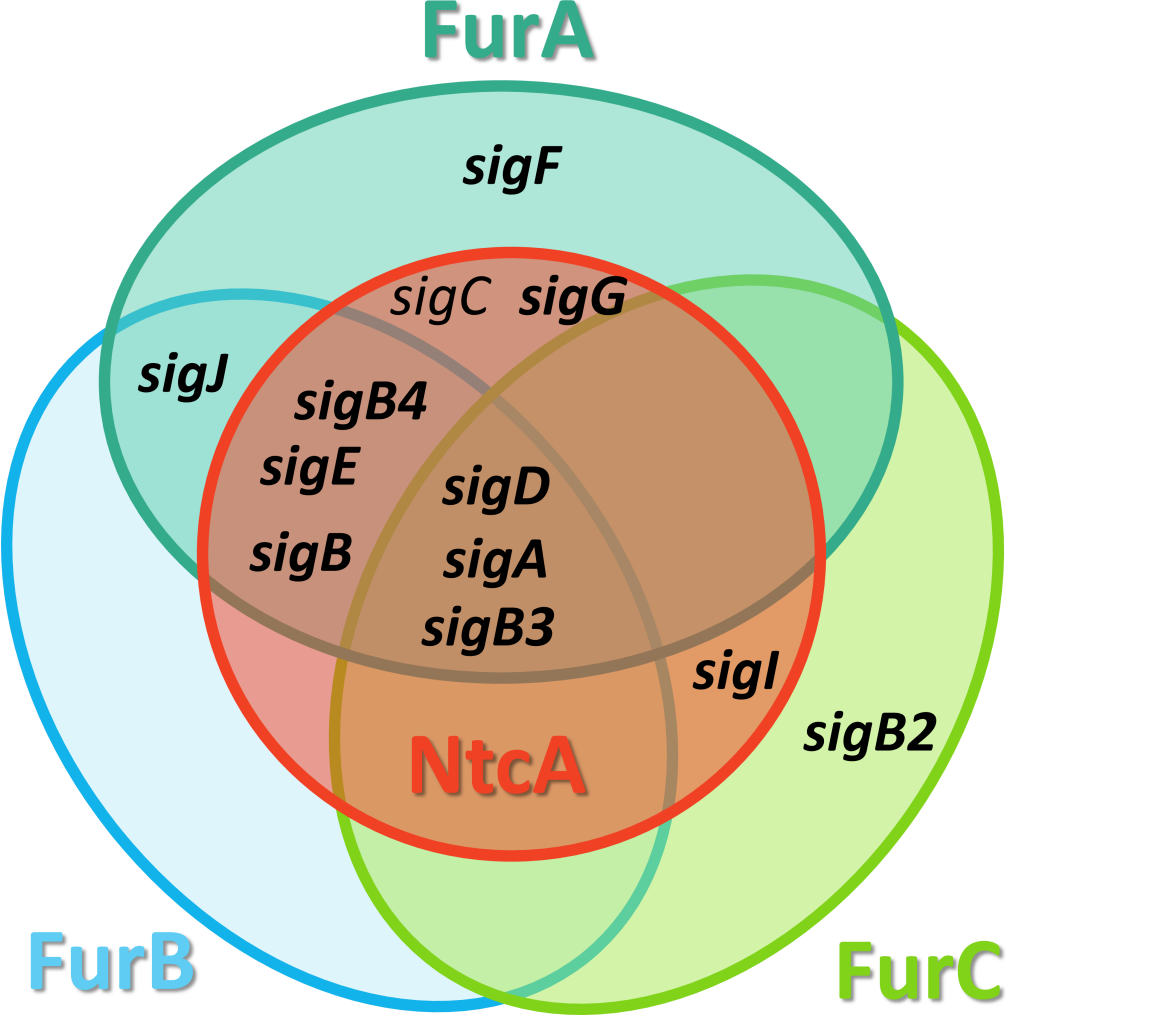
Venn diagram showing the coregulation of the sigma factors from *Anabaena* sp. PCC 7120 performed by FUR proteins and NtcA. Targets found in this work are indicated in bold.

As can be observed in the Venn diagram (Fig. 2) *sigA*, *sigD*, *sigB4*, *sigE*, *sigJ,* and *sigB3* are coregulated by FurA and FurB. All of them with the exception of *sigJ* are also controlled by NtcA, suggesting a high level of interconnection between these three networks. Furthermore, *sigD*, *sigA,* and *sigB3* are coregulated by FurA, FurB, FurC, and NtcA. The Venn diagram shows that all sigma factors from *Anabaena* sp. PCC 7120 are potentially controlled by at least one FUR paralog and that nine of them are also coregulated by NtcA.

### FUR proteins modulate the expression of all the sigma factors present in *Anabaena* sp. PCC 7120 and some of them are coregulated by NtcA

**Fig 3:**
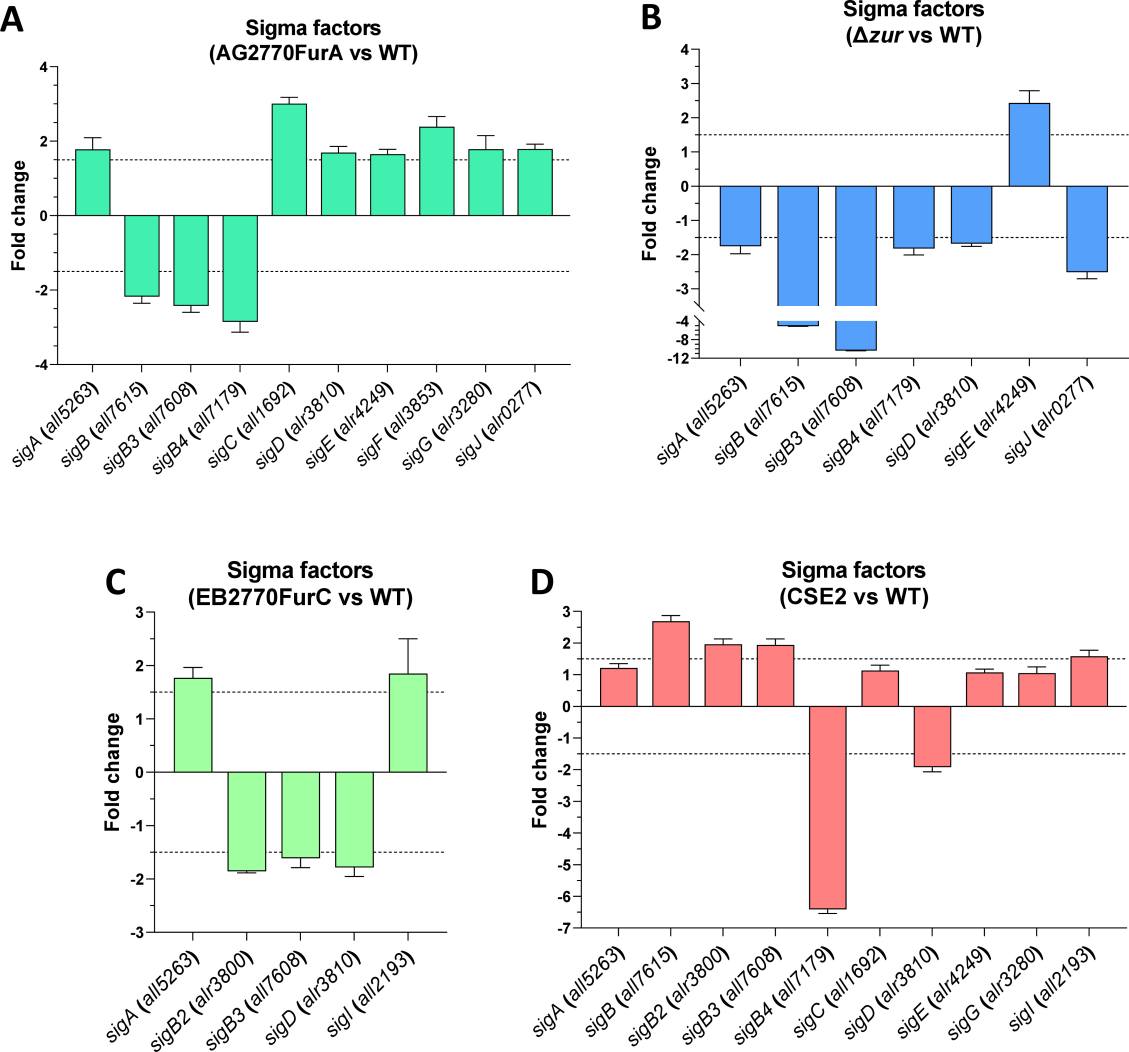
Influence of FUR and NtcA misregulation on the mRNA levels of sigma factor genes. (**A**) Levels of sigma factors mRNA in a *furA* overexpressing strain (AG2770FurA) vs the wild-type strain. (**B**) Levels of sigma factors mRNA in a *furB* deletion strain (Δ*zur*) v the wild-type strain. (**C**) Levels of sigma factors mRNA in a *furC*-overexpressing strain (EB2770FurC) vs the wild-type strain. (**D**) Levels of sigma factors mRNA in a *ntcA* deletion strain (CSE2) vs the wild-type strain. Relative real-time RT-PCR was used. Values are expressed as fold change and correspond to the average of three independent assays; the standard deviation is indicated.

Expression levels of sigma factors potentially controlled by FUR proteins were measured by real-time RT-PCR in their corresponding misregulation strain (*furA*-overexpression strain [AG2770FurA] for FurA, *furB*-deletion strain [Δ*zur*] for FurB, and *furC*-overexpression strain [EB2770FurC] for FurC) vs the wild-type strain. All of them changed their expression at least 1.5-fold, suggesting their direct modulation by these regulators (Fig. 3A through C). Finally, the mRNA levels of the nine sigma factors potentially regulated by NtcA were analyzed in a Δ*ntcA*-strain (CSE2). It was found that *sigB4* and *sigD* were downregulated in the CSE2 strain and, thus, would be potentially activated by NtcA, whereas *sigB*, *sigB2*, *sigB3,* and *sigI* were overexpressed and, thus, potentially repressed by this regulator (Fig. 3D). However, the activation or repression of sigma factors must be taken cautiously since compensatory effects could be happening between different sigma factors.

## DISCUSSION

In the last years, transcriptomic analyses of misregulation strains of FUR proteins clearly revealed that FurA, FurB, and FurC from *Anabaena* sp. PCC 7120 are master regulators. Global regulators exert their role directly on genes that are their direct targets, but they also perform an indirect and complex regulation involving other transcriptional regulators, two-component systems, serine/threonine kinases, and sigma factors.

**Fig 4:**
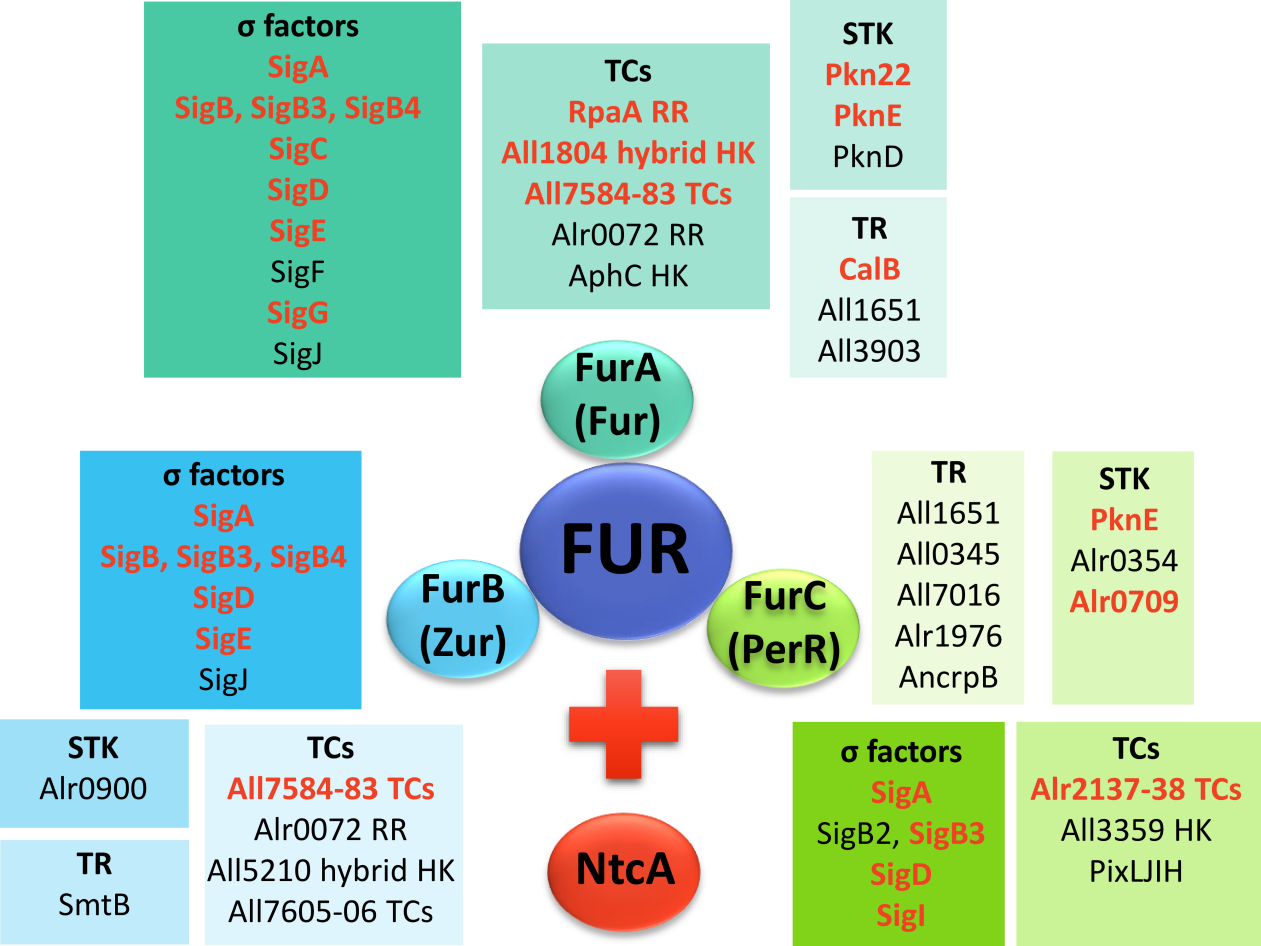
General scheme of regulatory networks performed by FUR proteins and NtcA. Genes coding for transcriptional regulators (TR), two-component systems (TCs), serine/threonine kinases (STK), and sigma factors (σ factors) directly regulated by FurA are shaded in turquoise tones, those regulated by FurB in blue tones, and those regulated by FurC in green tones. Genes whose expression is also regulated by NtcA are highlighted in red.

In this study, we found that three transcriptional regulators, five proteins from two-component systems, three serine/threonine kinases, and nine sigma factors are directly controlled by FurA in *Anabaena* sp. PCC7120 (Fig. 4). In other cyanobacteria such as *Synechocystis* sp. PCC 6803 and *Synechocystis* sp. PCC6714, the FurA regulon is more compact, with FurA-binding sites identified only upstream 33 protein-coding genes and the sRNA *IsaRI* (45). However, *Synechocystis* also exhibits secondary regulons mediated by other Fur-controlled regulatory proteins, such as Sll1408 (PcrR), Slr1489, and Sll1205 (PchR). Additionally, we found that in *Anabaena* sp. PCC7120, one transcriptional regulator, four two-component systems, one serine/threonine kinase, and seven sigma factors are directly regulated by FurB (ZUR) (Fig. 4). To the best of our knowledge, the Zur regulon in *Synechocystis* is unknown. However, analysis of the Zur regulon in *Synechococcus* PCC 7002 suggests that the set of Zur target genes is relatively small comprising only two clusters of genes, one of which is predicted to be involved in zinc transport, though no regulatory proteins were identified (46). Finally, we found that FurC (PerR) directly controls the expression of five transcriptional regulators, three two-component systems, three serine/threonine kinases, and five sigma factors (Fig. 4). In a *perR* deletion mutant of *Synechocystis* sp. PCC6803, 3 transcriptional regulators, 14 proteins related to two-component systems, 1 serine/threonine kinase, and 6 sigma factors showed altered expression. While it remains unclear whether these genes are directly regulated by PerR, it is highly likely that some of them contribute to the regulation of genes indirectly controlled by PerR (47). Most master regulator regulons have been obtained by using microarrays or RNA-seq analyses, either alone or in combination with *in silico* approaches. These studies typically compare differentially expressed genes in deletion or overexpression strains vs the wild-type strain. The number of regulatory proteins showing expression changes can vary depending on the complexity of the organism (e.g., *Anabaena* is more complex than *Synechocystis* or *Synechococcus*) and the relevance of the master regulator in the hierarchy of the cell. A key strength of our study is that, while we initially retrieved data on regulatory proteins potentially controlled by FUR proteins from RNA-seq analyses, we further validated these findings using EMSA. This approach allowed us to specifically identify the regulatory genes that are directly regulated by FUR proteins. When analyzing transcriptional changes in regulatory proteins from transcriptomic studies of FUR misregulation strains, we observe that, despite being directly regulated, some genes appear either upregulated or downregulated (Tables 1 to 3; Fig. 3). This fact suggests that the expression of certain regulatory proteins is either activated or repressed by FUR proteins, which aligns with previous studies defining FUR proteins as displaying a dual role as both activators and repressors (14, 16–18).

Interestingly, some regulatory genes with known function are regulated by more than one FUR family member (Table 4). These include the two-component system *all7584-83* that seems to be homologous to the *copS*/*copR* system, and *alr0072* (another homolog of *copR*), whose expression is regulated by both FurA and FurB. It has been described that FurA is involved in the homeostasis and transport of iron (48) but also of other metals such as zinc (15), whereas FurB is deeply involved in the regulation of metal trafficking in *Anabaena* sp. PCC 7120 (16). The direct regulation of the putative CopS/CopR system from *Anabaena* sp. PCC 7120 by FurA and FurB found in the present study suggests that both regulators could also be involved in the regulation of copper homeostasis.

In light of our results, it has also been found that both FurA and FurC directly regulate the expression of *pknE*. PknE is a serine/threonine kinase regulated by HetR, whose expression is required for the development of heterocysts under nitrogen starvation conditions (34, 49). The existence of a relationship between FurA, FurC, and nitrogen metabolism regulation was previously described (17, 18, 26, 43) so the fact that FurA and FurC are controlling *pknE* expression is another piece of evidence that reflects the involvement of these FUR paralogs in the regulation of this process.

Previous works revealed the existence of a cross-talk between FurA and NtcA regulatory networks (44). This was also observed in this work since *calB*, *rpaA*, *all1804, all7584-83, cyaD*, *cyaB2, all4379, cyaC, pkn22,* and *pknE* are regulated by the two master regulators (Table 4). However, here we report that FurB and FurC regulatory networks also overlap with NtcA since both *all7584-83* and *pknE* are also coregulated by NtcA (Table 4). Interestingly, the expression of most genes coregulated by FUR and NtcA is incremented under nitrogen starvation conditions (Table S2) (49), suggesting their involvement in the adaptation of *Anabaena* to nitrogen deprivation. It is also remarkable that the expression of the master regulator transducing the circadian clock output (*rpaA*) seems to be controlled by FurA and NtcA. This result has important implications since it connects iron and nitrogen homeostasis with the modulation of the circadian clock of *Anabaena* sp. PCC 7120. Indeed, the expression of *rpaA* increases 2.2-fold after 21 h of nitrogen deprivation (Table S2) (49). Anyway, more research must be done to clarify how this regulation works and the signals involved in the process.

**Fig 5:**
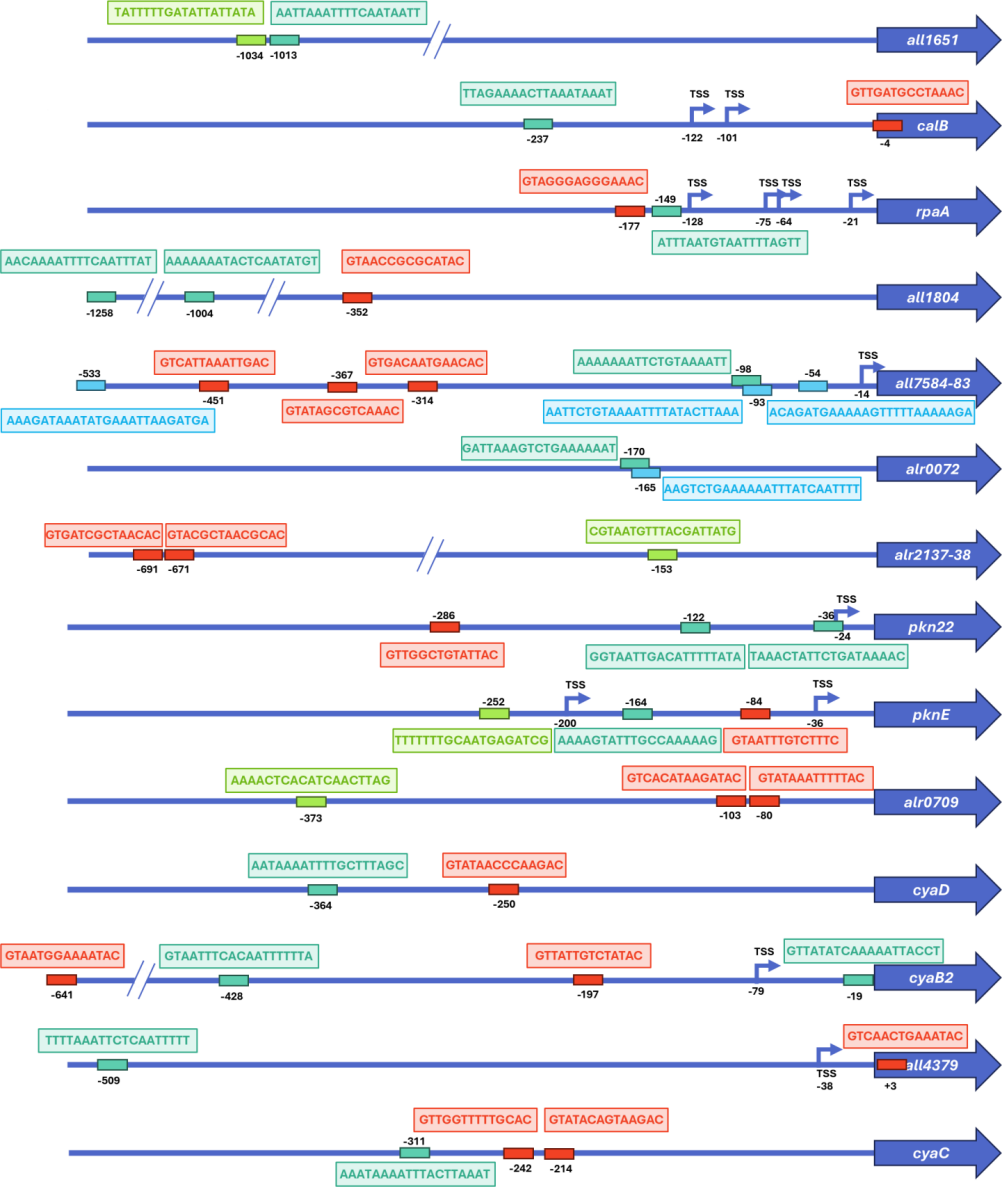
Graphical representation of the FurA, FurB, FurC, and NtcA boxes location in the promoter regions of the coregulated regulatory genes. FurA boxes are indicated in turquoise, FurB boxes are indicated in blue, FurC boxes are indicated in green, and NtcA boxes are indicated in red. Transcriptional start sites are indicated with an arrow and labelled as TSS. In all cases, the distance with respect to the start codon of the CDS is indicated.

We also studied the location of the putative FurA, FurB, FurC, and NtcA-DNA-binding boxes relative to the coding DNA sequence (CDS), the transcription start site (TSS), and −10 elements (when available) in the promoter regions of the coregulated regulatory genes (Fig. 5; Fig. S7). The regulation of these targets is complex, as multiple regulators may influence their expression. Additionally, as it was said before, FUR regulators can function as both repressors and activators, making it challenging to infer how the expression of these regulatory proteins is controlled. In addition, some TSSs have not been described, making the interpretation more complex. If we compare the location of the FUR/NtcA boxes (Fig. 5; Fig. S7) with the transcriptomic results (Table 4), we can infer some conclusions. In the case of *all1651*, *calB*, *all1804*, *alr2137-38*, *alr0709*, *cyaD*, *all4379,* and *cyaC*, the fact that the FUR/NtcA boxes are far from the gene and, therefore, from the TSS, may imply that, in these cases, these regulators are promoting the binding of RNA polymerase and would be in agreement with the fact that, according to transcriptomic data, these regulators are working as activators of these genes. On the contrary, when the DNA-binding box overlaps with the TSS and −10 box, such as in the case of the FurA-binding box in the *pkn22* promoter region, the regulator could be hampering the binding of the RNA polymerase to the TATA box and, thus, would explain why it is working as a repressor (26). In the case of *rpaA*, *all7584-83, pknE,* or *cyaB2*, the complexity of the number and positioning of the boxes, together with the existence of several TSSs in the case of *rpaA* and *pknE* promoters, makes it difficult to draw conclusions. Interestingly, we also found some cases in which two regulators might compete for promoter binding, such as one of the three FurB boxes in the *all7584-83* promoter or the single FurB box *alr0072*, which in both cases overlap with the FurA-binding box. In the case of *alr0072*, although the position of the TSS is unknown, the fact that FurB competes for FurA binding might explain why FurB is working as a repressor of its expression.

The fact that some regulatory proteins are common to both FUR and NtcA regulatory networks is intriguing. In recent years, we reported that FUR paralogs are involved in the regulation of heterocyst development and nitrogen fixation (17, 26, 43) so that this overlapping between both regulatory networks suggests that responses to nitrogen starvation are carried out by *Anabaena* integrating different signals such as iron availability (sensed through FurA and FurC) (50, 51), zinc availability (sensed through FurB and FurC) (51–53), and the redox status (sensed by FurA, FurB, and FurC) (54, 55). This signal integration is understandable because the development of heterocyst and nitrogen fixation are processes that do not only depend on nitrogen availability. For example, nitrogen fixation depends on nitrogenase activity, which requires both iron for nitrogenase functioning and absence of oxygen, that is determined by the redox status of the cell. Therefore, although NtcA is the master regulator of nitrogen metabolism, FUR proteins can be helping in the regulatory network of heterocyst development and nitrogen fixation.

Regarding sigma factors, it is important to note that FUR proteins regulate the expression of the 12 sigma factors of *Anabaena* sp. PCC 7120. FUR proteins are regulators that allow the adaptation of *Anabaena* sp. PCC 7120 to different stressful scenarios, so it is logical that they can modulate the transcription of determined sigma factors in response to a concrete stressful situation. Moreover, many sigma factors are regulated by more than one FUR paralog and are also coregulated by NtcA, evidencing again the interplay between these four transcriptional regulators. Sigma factors involved in the response to nitrogen starvation (*sigC*, *sigE,* and *sigG*) (11) are controlled by FurA (*sigC*, *sigE,* and *sigG*), FurB (*sigE*), and NtcA (*sigE* and *sigG*), a fact which corroborates the involvement of these three regulators in the regulation of this process. On the other hand, FurB modulates the expression of *sigJ*, which participates in the regulation of desiccation tolerance (12). This fact is in agreement with previously reported data in which it was observed that FurB controlled the expression of several desiccation-tolerance-related genes, including those involved in the synthesis of trehalose and the transference of saccharide moieties, among many others (16). Moreover, we found that *sigB2*, which regulates the early response of *Anabaena* to salt stress and whose expression is controlled by the response regulator OrrA (9), is also regulated by FurC. Finally, SigB and SigD factors were described as the central sigma factors of oxidative stress, heat, and high light acclimation in *Synechocystis* sp. PCC 6803 (56, 57). Although their functions remain unclear in *Anabaena*, in the present work, we found that their expression is regulated by the three FUR proteins, which are well known to control several processes for the adaptation of *Anabaena* to oxidative stress (14, 54, 58, 59).

In essence, FUR paralogs and NtcA are global regulators that bind directly to the promoters of their target genes, controlling their expression. However, they also exert significant indirect regulation by influencing the expression of other regulatory proteins, mainly transcriptional regulators, two-component systems, serine/threonine kinases, and sigma factors. In the present work, we contribute to the description of how FUR proteins perform this indirect regulation by identifying 8 transcriptional regulators, 10 proteins belonging to two-component systems, 6 serine/threonine kinases, and 12 sigma factors to be directly regulated by FUR proteins, forming a global regulatory network (Fig. 4). In addition, we have also analyzed the overlapping of FUR and NtcA regulatory networks, unveiling that several members of this regulatory network are coregulated by them, suggesting the cooperation of FUR and NtcA in the response to stress situations (Fig. 4). Further studies must be carried out to identify the direct targets of those transcriptional regulators, two-component systems, and serine/threonine kinases in order to be able to decipher the entire regulatory networks.

## MATERIALS AND METHODS

### Protein purification

FurA, FurB, FurC, and NtcA were purified as previously described by Pellicer et al. (60), Sein-Echaluce et al. (53), Sarasa-Buisan et al. (17), and Álvarez-Escribano et al. (61), respectively.

Recombinant His-tagged FurA, FurB, FurC, and NtcA proteins were produced in *Escherichia coli* BL21 cells transformed with pET-28a(+) plasmids (Novagen) containing the *furA* (*all1691*), *furB* (*all2473*), *furC* (*alr0957*), or *ntcA* (*alr4392*) gene sequences followed by a His-tag. Cell cultures were grown in Luria–Bertani (LB) medium at 37°C until late exponential phase (OD_600_ 0.6–0.7). Recombinant protein production was induced with 1  mM isopropyl β-D-1-thiogalactopyranoside (IPTG), and cells were harvested by centrifugation after 16 h of induction at 18°C.

For FurA and FurB purifications, 10 g of cells was resuspended in 50 mL of buffer 0.1 M NaH_2_PO_4_, 0.01 M Tris, 2 M guanidine–HCl, pH 8.0 (buffer A) supplemented with 1 mM of protease inhibitor phenylmethylsulfonyl fluoride (PMSF). After 10 cycles of sonication, cell debris was removed by centrifugation, and the resulting supernatant was loaded onto a Chelating Sepharose Fast Flow column previously loaded with 0.25 M ZnSO_4_. The column was washed with 0.5 M (NH_4_)_2_SO_4_ in buffer A and then with 35 mM glycine in buffer A, and FurA or FurB was subsequently eluted in a linear gradient of 0–1 M imidazole in buffer A. The resulting fractions were analyzed by SDS-PAGE, and the purest fractions were dialyzed against 10  mM acetic acid/acetate buffer pH 5.5 to be stored at −20°C.

For FurC purification, 10 g of cells was resuspended in 50 mL of buffer 50 mM Tris-HCl pH 7.5 in the presence of 10 mM EDTA (buffer A) to avoid metal catalyzed oxidation and supplemented with 1 mM of protease inhibitor phenylmethylsulfonyl fluoride (PMSF). After 10 cycles of sonication, cell debris was removed by centrifugation, and the resulting supernatant was loaded onto a 20 mL Heparin Sepharose column equilibrated in buffer A. The column was washed with buffer A supplemented with 0.1 M NaCl, and FurC was eluted in a linear gradient of 0–0.5 M NaCl in buffer A. FurC-containing fractions were pooled and dialyzed in 50 mM Tris-HCl pH 7.5, and the resultant FurC was further subjected to an ion exchange chromatography on a 20 mL DEAE cellulose column equilibrated with 50 mM Tris-HCl pH 7.5. The column was washed with 50 mM Tris-HCl pH 7.5, 0.1 M NaCl, and protein was eluted using a linear gradient 0.1–0.7 M NaCl in 50 mM Tris-HCl pH 7.5. The resulting fractions were analyzed by SDS-PAGE and spectrofotometrically, and the purest fractions were pooled and dialyzed in 50 mM Tris-HCl pH 7.5, 150 mM NaCl to be stored at –80°C.

For NtcA purification, 10 g of cells was resuspended in 50 mL of buffer 20 mM sodium phosphate buffer (pH 7.2) containing 500 mM NaCl, 20 mM imidazole supplemented with 1 mM of protease inhibitor phenylmethylsulfonyl fluoride (PMSF). After 10 cycles of sonication, cell debris was removed by centrifugation, and the resulting supernatant was loaded onto a Chelating Sepharose Fast Flow column previously loaded with 0.25 M NiSO_4_. The column was washed with 40 mM imidazole in buffer A, and NtcA was eluted in a linear gradient of 0–1 M imidazole in buffer A. The resulting fractions were analyzed by SDS-PAGE, and the purest fractions were dialyzed against 20 mM sodium phosphate buffer, 500 mM NaCl, 10% glycerol, pH 7.2 to be stored at −20°C.

### Electrophoretic mobility shift assays

Promoter regions used for EMSA analyses consisted of 250–450 bp DNA fragments and were obtained by PCR, using *Anabaena* sp. PCC 7120 genome as template and the primers included in Table S1. For some FurA potential targets analyzed in this work, the presence of FurA boxes was predicted in previous works (14). Consequently, in these cases, we selected EMSA regions of 250–450 centered around the predicted FurA boxes. In the case of NtcA targets, we analyzed the binding of this regulator to the promoter region of genes that were found as potential NtcA targets in ChIP seq analyses (20), so the region selected for EMSA was centered around the NtcA-binding region found in the ChIP seq. For the rest of the genes, we considered that in bacteria, the majority of the putative TSSs are located between 20 and 40 nucleotides from the start codon, while most of the transcription factor binding sites are located between −250 and +50 nucleotides from the TSS (62). Consequently, we selected regions between positions −350 and +50 with respect to the translational start codon.

Reactions for EMSA analyses were performed by mixing increasing concentrations of purified FurA, FurB, FurC, or NtcA in a final volume of 20 µL with 50–100 ng of DNA fragments in a binding buffer containing 10 mM Bis Tris-HCl, pH 7.5, 40 mM KCl, 0.1 mg/mL BSA, 1 mM of 1,4-dithiothreitol (DTT), and 5% (vol/vol) glycerol. In the case of FurA and FurC, 100 µM of MnCl_2_ was added to the binding buffer, whereas in the case of FurB, 5 μg of ZnSO_4_ was added to the binding buffer. To probe non-specific binding, a competitor DNA was used, which consisted of 50–100 ng of a 150 bp internal fragment of gene *pkn22* (if*pkn22*). Samples were incubated at room temperature for 30 min, mixed with a 6 × loading buffer (containing 30  mM Bis-Tris pH 8, 30% glycerol, and 0.05% bromophenol blue), and loaded into a non-denaturing 6% polyacrylamide gel. Electrophoresis was run at 4°C under a voltage of 90 V for approximately 110 min, and in the case of FurA and FurC, both gel and running buffer included 100 µM MnCl_2_. Gels were stained with SYBR Safe (Invitrogen) and visualized in a GelDoc 2000 device (Bio-Rad).

### Bacterial strains and culture conditions

Cyanobacteria strains used in this work were the wild-type *Anabaena* sp. PCC 7120, as well as the genetically modified strains AG2770FurA, Δ*zur*, EB2770FurC, and CSE2. The AG2770FurA strain is a *furA*-overexpressing strain containing the pAM2770*furA* plasmid, which harbors the *furA/fur* gene (*all1691*) downstream the copper-inducible *petE* (plastocyanin) promoter (63). The Δ*zur* strain is a deletion–insertion mutant with a C.S3 cassette interrupting the *furB/zur* gene (*all2473*) (52). The EB2770FurC strain is a *furC*-overexpressing strain containing the pAM2770*furC* plasmid, which harbors a *furC/perR* gene (*alr0957*) downstream the copper-inducible *petE* (plastocyanin) promoter (54). The CSE2 strain is an insertional mutant of the *ntcA* gene (64).

All cyanobacterial cultures were grown photoautotrophically in BG11 at 28°C under constant illumination of 30 μE m^−2^ s^−1^ in an orbital shaker at 130 rpm. Cultures of strains AG2770FurA and EB2770FurC contained 50 µg mL^−1^ of neomycin (Sigma-Aldrich), whereas cultures of Δ*zur* and CSE2 contained 2 µg mL^−1^ of streptomycin and 2 µg mL^−1^ of spectinomycin (Sigma-Aldrich). As the copper concentration present in BG11 has been described to be sufficient to drive overexpression from the *petE* promoter, no exogenous copper was added neither to the AG2770FurA nor to the EB2770FurC cultures (63).

### RNA extraction

The RNA samples used for differential gene expression analyses were extracted and purified as described in Sarasa-Buisan et al. (17). Three independent cultures of each strain were set up by diluting cells from late exponential phase cultures to an OD_750_ of 0.3 in a final volume of 100 mL in fresh BG11. Cultures were incubated in 250 mL Erlenmeyer flasks on an orbital shaker at 130 rpm and 28°C under a continuous light regime of 30 µmol photons m^−2^ s^−1^_,_ and samples were grown for 6  days to an OD_750_ of 0.6–0.7. RNA was extracted and purified from 25 mL of each culture following the method described in Sarasa-Buisan et al. (17).

### Real-time RT-PCR assays

The pool of cDNA was synthesized by reverse-transcription of 2 µg of total RNA using SuperScript retrotranscriptase (Invitrogen) following the manufacturer’s conditions. Real-time PCR was performed using the QuantStudio 5 system (Applied Biosystems). Each reaction was set up by mixing 12.5 µL of SYBR Green PCR Master Mix with 0.4 µL of 25  µM primer mixture and 10 ng of cDNA template in a final volume of 30 µL in nuclease-free water (Ambion), with additional water added instead of cDNA for negative controls. Amplification was performed at 60°C for 40 cycles. The sequences of specific primers of selected genes were designed with Primer Express 3 (Thermofisher) and are given in Table S1. Transcript levels of target genes were normalized to those of the housekeeping gene *rnpB* (65). Relative quantification and expression fold changes were calculated according to the comparative Ct method (ΔΔCt method) (66), where the fold change threshold was set up to ≥1.5-fold or ≤−1.5-fold.

## Data Availability

All data can be found within the article and its supplemental material.

## SUPPLEMENTAL MATERIAL

Supplemental Figures msystems.00373-25-s0001.docx Figures S1 to S7.

Supplemental Tables msystems.00373-25-s0002.docx Tables S1 and S2.

ASM does not own the copyrights to Supplemental Material that may be linked to, or accessed through, an article. The authors have granted ASM a non-exclusive, world-wide license to publish the Supplemental Material files. Please contact the corresponding author directly for reuse.
