# Supplementary material for: Regulatory networks of FUR and NtcA are intertwined by transcriptional regulators, two-component systems, serine/threonine kinases, and sigma factors in Anabaena sp. PCC 7120
Source: mSystems. 2025 Jun 25;10(7):e00373-25. doi: 10.1128/msystems.00373-25 (PMC12282138; doi:10.1128/msystems.00373-25)
Supplement: Supplemental Figures — Figures S1 to S7. [file msystems.00373-25-s0001.docx]

**SUPPLEMENTARY FIGURES**

**Figure S1. EMSA assays showing the ability of FurA to bind *in vitro* to the promoter regions of novel direct target genes.** DNA fragments free or mixed with increasing concentrations of recombinant FurA (nM) were separated by 6 % PAGE. An internal fragment of the gene *pkn22* was used as non-specific competitor DNA. **A.** Promoter regions of transcriptional regulators **B.** Promoter regions of two-component system proteins **C.** Promoter regions of serine/threonine kinases. **D.** Promoter regions of other genes with regulatory functions.

**Figure S2. EMSA assays showing the ability of FurB to bind *in vitro* to the promoter regions of novel direct target genes.** DNA fragments free or mixed with increasing concentrations of recombinant FurB (nM) were separated by 6 % PAGE. An internal fragment of the gene *pkn22* was used as non-specific competitor DNA. **A.** Promoter regions of two-component system proteins **B.** Promoter regions of serine/threonine kinases.

**Figure S3. EMSA assays showing the ability of FurC to bind *in vitro* to the promoter regions of novel direct target genes.** DNA fragments free or mixed with increasing concentrations of recombinant FurC (nM) were separated by 6 % PAGE. An internal fragment of the gene *pkn22* was used as non-specific competitor DNA. **A.** Promoter regions of transcriptional regulators **B.** Promoter regions of two-component system **C.** Promoter regions of serine/threonine kinases.

**Figure S4. EMSA assays showing the ability of NtcA to bind *in vitro* to the promoter regions of regulatory genes controlled by FUR proteins.** DNA fragments free or mixed with increasing concentrations of recombinant NtcA (nM) were separated by 6 % PAGE. An internal fragment of the gene *pkn22* was used as non-specific competitor DNA.

**Figure S5. EMSA assays showing the ability of FUR proteins to bind *in vitro* to the promoter regions of sigma factors from *Anabaena* sp. PCC 7120.** DNA fragments free or mixed with increasing concentrations of recombinant FurA, FurB or FurC (nM) were separated by 6 % PAGE. An internal fragment of the gene *pkn22* was used as non-specific competitor DNA. **A.** EMSAs performed with FurA **B.** EMSAs performed with FurB **C.** EMSAs performed with FurC

**Figure S6. EMSA assays** **showing the ability of NtcA to bind *in vitro* to the promoter regions of sigma factors from *Anabaena* sp. PCC 7120.** DNA fragments free or mixed with increasing concentrations of recombinant NtcA (nM) were separated by 6 % PAGE. An internal fragment of the gene *pkn22* was used as non-specific competitor DNA.

**Figure S7.** **Analysis of the putative FurA, FurB, FurC and NtcA-DNA binding boxes location with respect to the coding DNA sequence (CDS) and the transcription start site (TSS) and -10 elements (when known) in the promoter regions of the coregulated regulatory genes.** Predicted FurA, FurB, FurC and NtcA binding sites are indicated in turquoise, blue, green and red, respectively. TSS are indicated in bold and red, CDS regions are indicated in bold and blue, -10 elements are represented in bold and underlined. boxes FurA boxes were predicted using the weight matrix from Gonzalez *et al.* (15). FurB boxes were predicted using the weight matrix from Napolitano *et al.* (52). FurC boxes were predicted using the weight matrix from Sarasa-Buisan *et al.* (18). NtcA boxes were predicted using the weight matrix from Picossi *et al.* (20). TSS and -10 elements were annotated according to Mitschke *et al.* (67).

67. Mitschke J, Vioque A, Haas F, Hess WR, Muro-Pastor AM. 2011. Dynamics of transcriptional start site selection during nitrogen stress-induced cell differentiation in *Anabaena* sp. PCC7120. Proc Natl Acad Sci USA 108:20130–20135. <https://doi.org/10.1073/pnas.1112724108>

**Figure S1. EMSA assays showing the ability of FurA to bind *in vitro* to the promoter regions of novel direct target genes.** DNA fragments free or mixed with increasing concentrations of recombinant FurA (nM) were separated by 6 % PAGE. An internal fragment of the gene *pkn22* was used as non-specific competitor DNA. **A.** Promoter regions of transcriptional regulators **B.** Promoter regions of two-component system proteins **C.** Promoter regions of serine/threonine kinases. **D.** Promoter regions of other genes with regulatory functions.


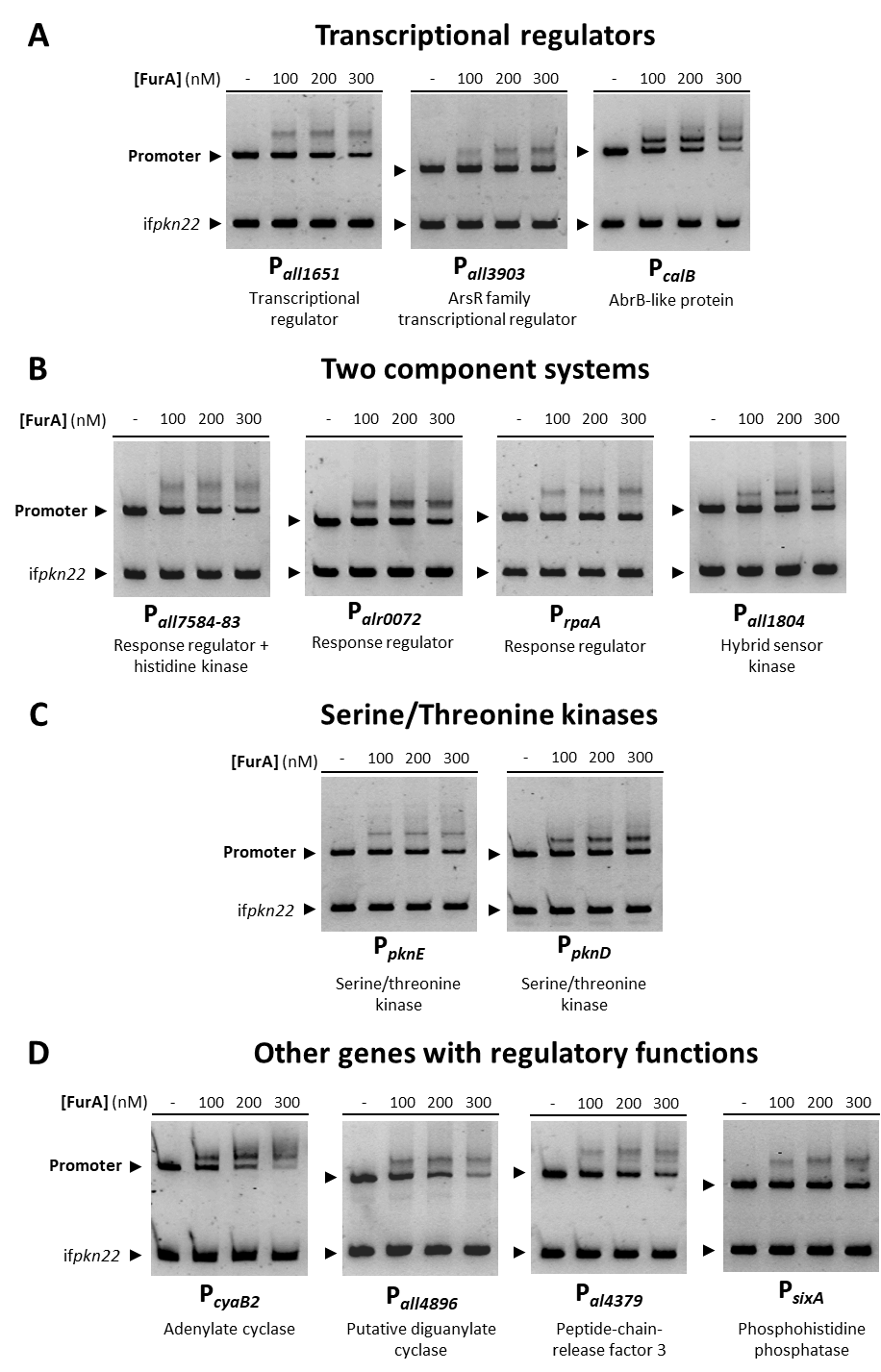


**Figure S2. EMSA assays showing the ability of FurB to bind *in vitro* to the promoter regions of novel direct target genes.** DNA fragments free or mixed with increasing concentrations of recombinant FurB (nM) were separated by 6 % PAGE. An internal fragment of the gene *pkn22* was used as non-specific competitor DNA. **A.** Promoter regions of two-component system proteins **B.** Promoter regions of serine/threonine kinases.


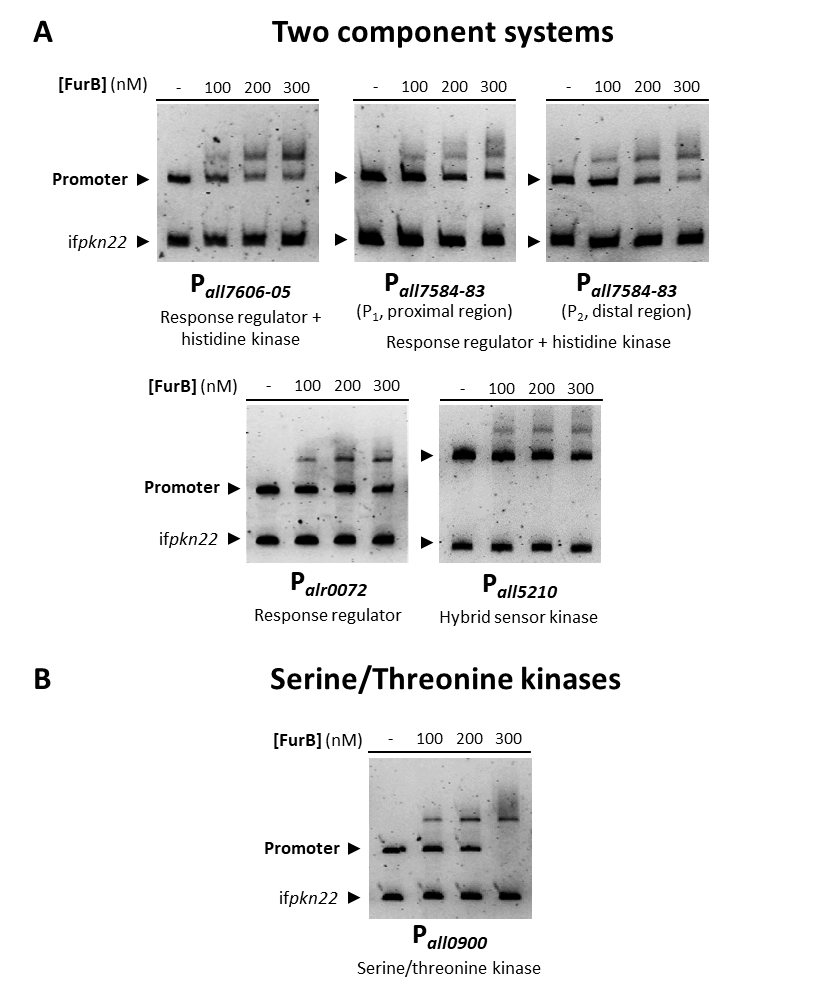


**Figure S3. EMSA assays showing the ability of FurC to bind *in vitro* to the promoter regions of novel direct target genes.** DNA fragments free or mixed with increasing concentrations of recombinant FurC (nM) were separated by 6 % PAGE. An internal fragment of the gene *pkn22* was used as non-specific competitor DNA. **A.** Promoter regions of transcriptional regulators **B.** Promoter regions of two-component system **C.** Promoter regions of serine/threonine kinases.


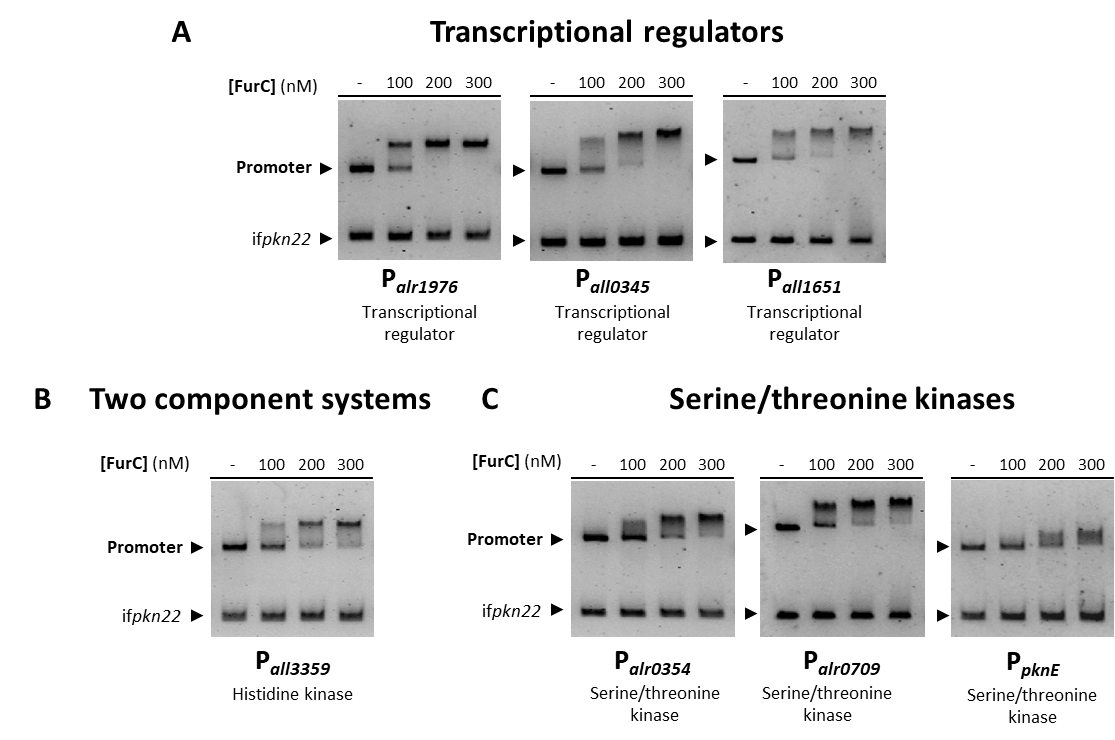


**Figure S4. EMSA assays showing the ability of NtcA to bind *in vitro* to the promoter regions of regulatory genes controlled by FUR proteins.** DNA fragments free or mixed with increasing concentrations of recombinant NtcA (nM) were separated by 6 % PAGE. An internal fragment of the gene *pkn22* was used as non-specific competitor DNA.


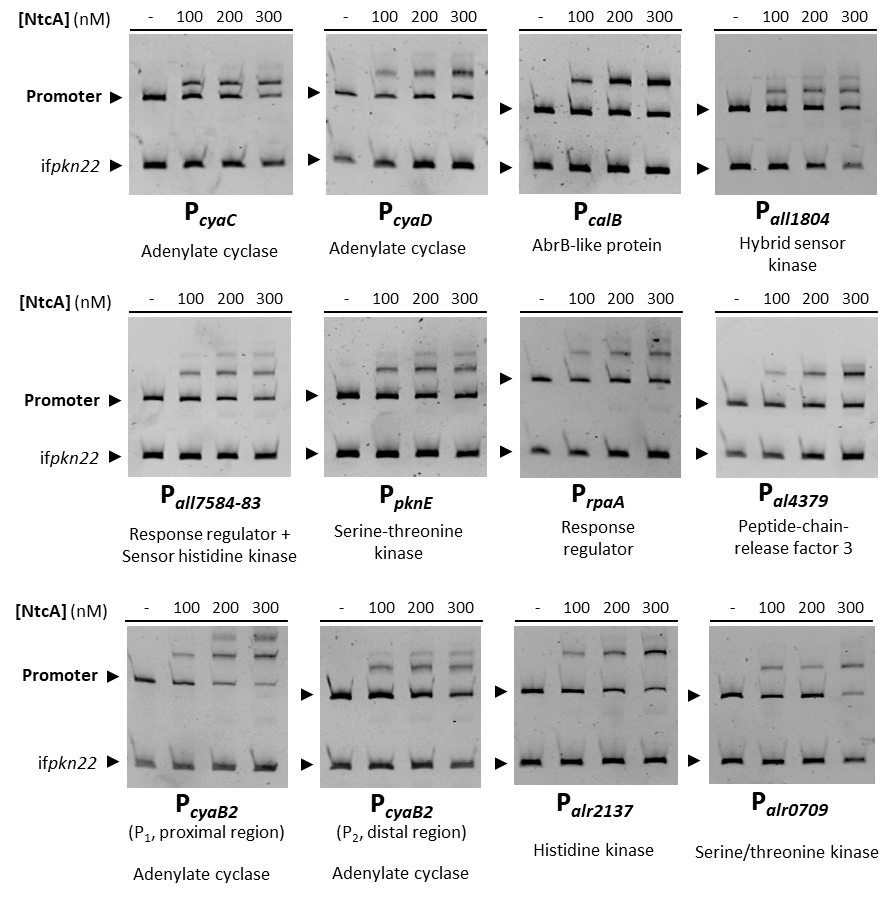


**Figure S5. EMSA assays showing the ability of FUR proteins to bind *in vitro* to the promoter regions of sigma factors from *Anabaena* sp. PCC 7120.** DNA fragments free or mixed with increasing concentrations of recombinant FurA, FurB or FurC (nM) were separated by 6 % PAGE. An internal fragment of the gene *pkn22* was used as non-specific competitor DNA. **A.** EMSAs performed with FurA **B.** EMSAs performed with FurB **C.** EMSAs performed with FurC


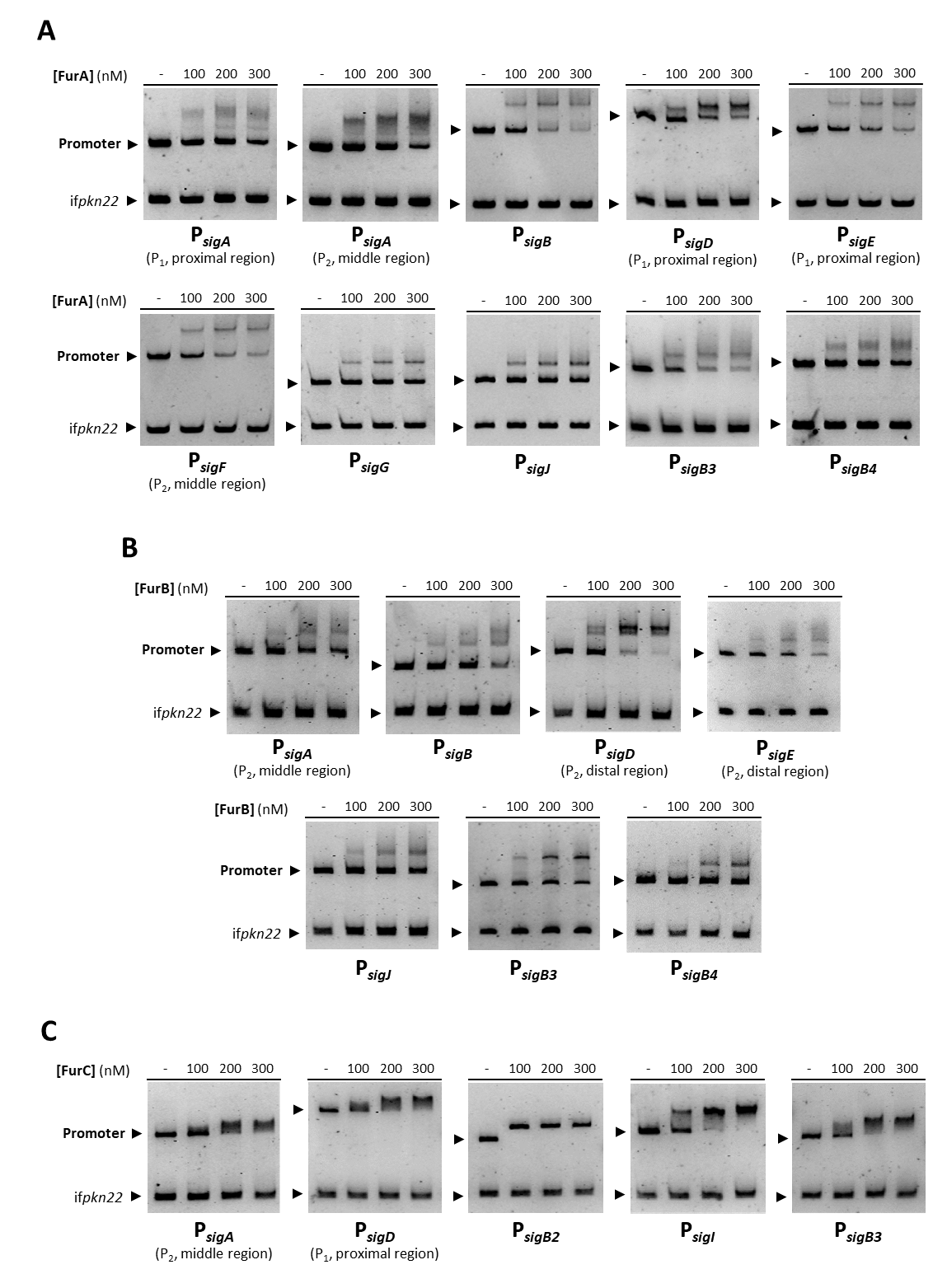


**Figure S6. EMSA assays** **showing the ability of NtcA to bind *in vitro* to the promoter regions of sigma factors from *Anabaena* sp. PCC 7120.** DNA fragments free or mixed with increasing concentrations of recombinant NtcA (nM) were separated by 6 % PAGE. An internal fragment of the gene *pkn22* was used as non-specific competitor DNA.


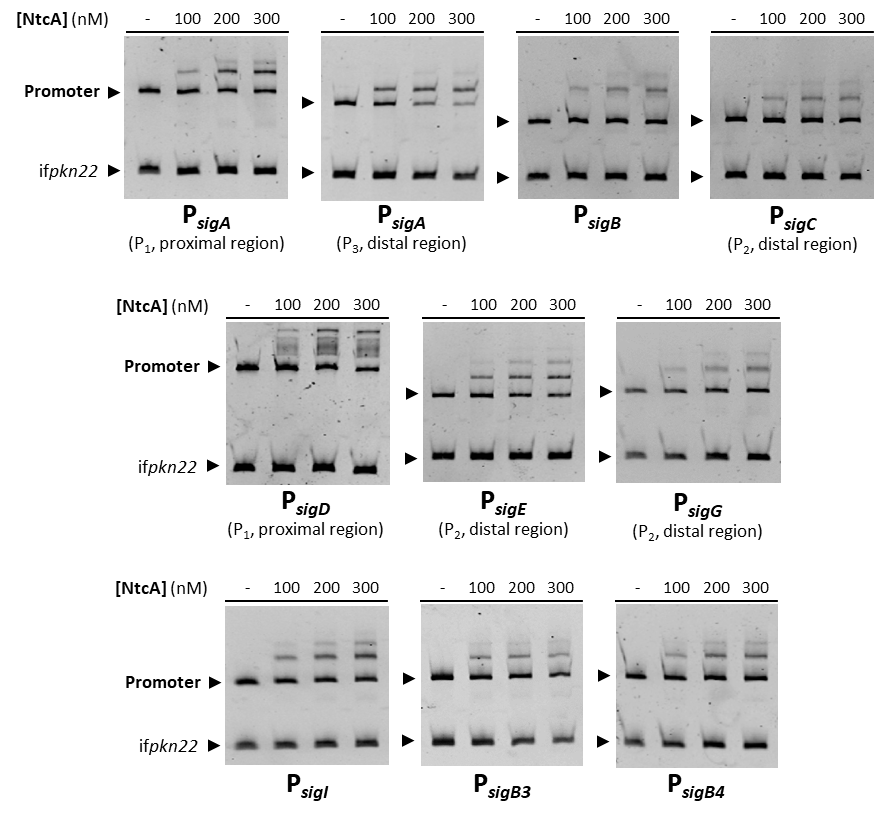


**Figure S7.** **Analysis of the putative FurA, FurB, FurC and NtcA-DNA binding boxes location with respect to the coding DNA sequence (CDS) and the transcription start site (TSS) and -10 elements (when known) in the promoter regions of the coregulated regulatory genes.** Predicted FurA, FurB, FurC and NtcA binding sites are indicated in turquoise, blue, green and red, respectively. TSS are indicated in bold and red, CDS regions are indicated in bold and blue, -10 elements are represented in bold and underlined. boxes FurA boxes were predicted using the weight matrix from Gonzalez *et al.* (15). FurB boxes were predicted using the weight matrix from Napolitano *et al.* (52). FurC boxes were predicted using the weight matrix from Sarasa-Buisan *et al.* (18). NtcA boxes were predicted using the weight matrix from Picossi *et al.* (20). TSS and -10 elements were annotated according to Mitschke *et al.* (67).

**Legend**

| NNNNNNNNNNNNNNNNNNN | **FurA Box** |
| --- | --- |
| NNNNNNNNNNNNNNNNNNN | **FurB Box** |
| NNNNNNNNNNNNNNNNNNN | **FurC Box** |
| NNNNNNNNNNNNNN | **NtcA Box** |
| **NNNNNN** | **-10 element** |
| **N** | **TSS (Transcriptional Start Site)** |
| **ATGNNNNN** | **CDS (coding DNA sequence)** |

***all1651; transcriptional regulator***

CATTTATTTTTTTCAGCATTTAATAGTCCCTTAATTTTTTCCAATTTTCAGAACTTGCCACAGAACTACAAGAGGATGTTTGAAAAGTTCTTTTGTTTGTGGCGAAACTGTTTAGACCCGATCTAAGAGGATGTTTGAAAAGTGATTGGATGTGATTTTAAGCACTCGTTGATCCCCCCTAACACCCCTTAATAAGGGGGTAAAATGAATCAAAGTCCCCCTTAAAAAGGGGGATTTAGGGGGATCTAAAATGTTTTGCTACCAAGCATGGGACTTTTCAAACATCCTCTAAGTTGTGAGCCTTAACTGAACTGTATTAACTTACTTACAATGTAGATTTCCTGCAACAGTTATTATTTGTGACTTGAGTTTAGTTTTTTTTAAATGCCCATACAATGGATTATTTTCTGCCAAAAATGGAGATTTTTTATGTAGAATTAGACGCAAGTCTTGTGCTGTAATTTGTAGAGTTCCAATCAGAGGAGAAAAAGGTAACTACCGTGTACAACGTAACAAATTTAACCGCAAATCTTCAGTTTAATTGTTTGTTACATATTTGTAAATTTTTTCCGCTACTCACTTAGTCATCACCGTTGCCATCTCTATACTTTCATTTCATATCACTGAATATCAAAAGTTACCAATACCTAGATATAGGAATCTGATTTGATTTCTGTTGGCGTAGCCTGCACTAGCGCATAAAATTCTCAGTATCTGTAGGGTGGGCATTGCCCACCGGCTCATAGTTTTGGTGGGCAATGCCCAACGCCACCTGCTCCAAGCCGGGAAACCCGTCCAACGCAGTGGCTCCCCTACGTGTATTTCAAAAATCAAGTATGAGTCCTATAGTTTTTTTAAACGTTTTTACTAGCACACTCATGTGGTTTCAAAATTACCTTGTTTGCAGTTTTTATGATTTATTTTTTCCCGAAACATAAACATAAACTATCTATTTTTGATATTATTATATAAATTAAATTTTCAATAATTTAATTTGTCAATTAAAGATTTTTGATTATAGATTACAAATCTAAACAAAGCACTTCATGTATATAAATTAATTTCGACTTAAATTATTTATAAATAAGATTTAATATTAAACCAATAAGCTACTTCATGGTGAAAGAAAAAGCATAATTCCTAAATTAACACTATATGCAGCTTTTTCTTAAACCTAACCCCCAATCCCTTTCCTACAAGCGAAAGGGAGTAATAATCAAAGCCTCTGAGTAGAACAACTCAAAAAAACGAACTATGTGAAGGTAAATAAATACATCTTCCTTGATATTTGCGATCGCTCTGTTGCCCTTACTCATTGGGGTTTAGACGAGTTCACAGTGCGAACTAGACTACATCCCATCAATTCGATTGTTAAGTAATGTAACAGAACGACATAAAGATTAGTATGTTTATTGATGAATAAATACACGGAGAGGACTCACGCACTTTATAAATAGACAGCCTACAGTAAAGTACCGCGTTGGGTTTATAAGCTTTCTATTGCCATCTCTTCTCACTTCTTCTGCCCCTTTAGTGTTAGTTCATAAGCTAATCAGAGATGATTTATAGAGTAAACCTTAAATTTGTAATGGCGTGTTAAAGCAAAGCATAACGCACCACTATCATACGGTACGTTACGGCTATATATAATTTGCTCACACACCCAAATCCTTACACAACCGTAACACACCCTACTTAATATTCACTTTATAAATGGTCTCTGCAACTGACATCTATAAAGTATGTATGACACAACCCGCATAATCAGACTTTCGTCCATGTATGAATCATACTTGCTGTAAAGGCTCACATTTATTAACAGCAAAATTATATTTTTATGTAATAAGATTGCATAAAAACTACTTTACACCCTGAATAATATAGATAAAAGCAACAAATTTACGACCTGACAATCTGGAAGAAATTTATTTCCACCCAAAGTCCAAATTCCATCTCAAACTATCAATTAGTAAAAATTGACATT**ATGTTTTCCAACCAAAATATTTCCTCTACCAA**

***all2080; transcriptional regulator AbrB, CalB***

CTTAAGTCCAGCAAAGCCTTTACACTATAGTGTCCTTTGGTTGTTAGTTTCATTTTGTTAGTTGTCAATTGTCAGTTGTCAGTTGTTTTTTCATTACCAATGACTAATGACCAATGACTAAGGATCAGTTTTACTTATCATTTTTCTCCTTAACAATCAAGCCGACTAAGGAGACAACTTTTTACTCGACTTTAGAAAACTTAAATAAATATAGTTTAGCAGTGATTGATTAACTATATATAGTTATCCCGATTATCAAAATTAAATTGTCTTGCAAATATTGGGAATTGTGC**AACAAT**TTTGCTT**A**TGAGTGTAA**TATACT**TGGCT**A**GGCTGAGATAAAAACTAAAGGATATTGTTCCTATTAGCTCAACGTCAGC**TAAAAT**AAAATC**G**ATTCAACTACTTAAACCCCTGATTTTTGACCTAAGTTG**ATGCCTAAACAGAAAAAAATAGAACCCCTAGTCGGTGAAGAACTGCTCAAAAAAGTCAAAGAGCTAGAGAACCTTA**

***all0129; two-component response regulator; RpaA***

GCGGATTGTAGGGAGGGAAACATATCATACTGCGAATTTAATG**TAATTT**TAGTTTT**G**TTTTCTTCATACTAGTAGTTTTAAGGTTTATGTTAGATT**TAAAAT**CTTTTT**TATAAT**CAAGAC**A**GAAATCTTTGTATCTACCAAAGGTTCTTATT**TATCCT**TAATA**A**GTAGTAATAATTTTTTAAGA**ATGCCAAGGATTCTTGTAATAGACGATGACCC**

***all1804; two-component hybrid sensor and regulator***

ATTTGCAAGGCATCGCTACGTAAATCCATGTTTTCACTCCTCTGTTGATTTTTACACCACATCCCACTACCAGTTTTTTGATAATACTGGATATTGTTTTGACTAAACTGGAAAAACTAGAGATTAGCTAGTATTGCTCCTAAATATTAATGTTTTTTTGTATCAAAATTAACAAAATTTTCAATTTATTAAAATCACATCTATTTTGATGTGATTTGTGCTGTACGTATTTGCGTTGTTGTTTGATATTACATGATAAATTCTTGGTGTAAATATAAACAGTGATAGCAATGTGACATTCTGTAATATACCAAATTTATCTTGTTATTCAAAGTTAATTCCCCAGTATCGAGTCTTAAAAGTCCCTGTGGCAGAGATTAATTTTTAGATAACGACTACGTGCAACCTTAAGATATTGATAAACAAAAAAATACTCAATATGTGTGATGTGTCAGTTAGAAAAACTGTGGACGCACCGAAAAATTATTATTTATGTAACCATCTAAGAGGCTATTTTAAAAGTATTTTTGTCTGTATAGAAAACCTTTTTTCTTCGTTAAATATTATTTAAAATTAGTATTTACTGTCTGTTCAATGGGGATTAGAAACTATTTAGAAAATAAATAAAAAACTATATTTATTAGATAGATAAAGATATGAGATTGTCTTTCTTGTTCAAGATATATGAATTTGATTTAATCATTGTATATAATGACGTATCTATAGTGTGTGTATAGCCCGTACTATATATAATTCAAAAATCAAATAGTAGTCCTATATAACCTTTGGTGTAGGGAATATTATAAATTTTGAATACTGCATAATTAATTTTTATTTAACGTCTATCAAGGGCTTTCAATTTGTTTACATCAGTTGAGAAGAATATGTTAAAAATGAATTTCTGTTCATTTATTTATATTCATAGAAGCACTTTACCAGGACTAATTAGAAGTGCAAAAACAAAATGGTAAAGATTTTAAGTGTTAACAGCATTTAAGAAGATTTTAATTACTGACACAGCAAGATATATATATAGATAGATGTAAACCAATACTTTTGGCAGTAGTTTAAATCAGGTAACCGCGCATACCGATAGTAAATCATCCTAAACATTACAATCGTATAGGAATCCGATTTGATTATTGTTGGCGTAGCCTGCGCTTGTGTATAAAAATCCAAGTATCTAGTCCACTTGCTCCAAGCAGGGAACCTCCTTCTCAACGTTCGCAGAGCGCAGCGTTGCCTAGCAAAGACACTCCTCCAGAGAAGCAAGCGTTCGACTAATCTCACGCCGAAGTCTACCCGTAGCGTCCCCCAGGGAACGTAGAGAGGATCGGACTGCCTAGCTTACCAACGCAGTGGCTCCCCTACGCATAATTCAAAAATCAAATAGTAGTCCTATATATAAATAAAGTTACAAAAGCCTCA**ATGTTAGAATTC**

***all7584; two-component response regulator (Operon all7584-83)***

ACAGGTTTATTTTTTCAAGCCAATTCTCAGAAATGCCTGTATTCATTCATGAATAGACTCGATATTTCTATCCTGAAAGATAAATATGAAATTAAGATGAAATTTCAGATATTGTCTTAAATAATTTCCAAAAATCAGTAGCTATGATTTTCTCATTGTCATTAAATTGACTTCCATGATTTGCTTTGTATACACTATTGCTTGCATGACGATGAAGCTTAAGTTAATGGAGGGTTTGTTTGTATAGCGTCAAACTCGAATATGAGAAAAATAATGCTAATGAACTTTCTTGATGTGACAATGAACACCAATAGTTTATAAATCAGTTTATAAATTTTCGGGAAATAGCTGGAATTTCATCTTAGTTTCATCTTTACCTGTCAAAATAAATAGTGACTCAATAGACTCTTGCAAATACTTAGTTTCTGTAACTCCTCAAACTAAGACCACACCTATTTTGGCAAGTCAATCTACTGAGTCCTTTTTCACTGTTAACATTTTGCTATTTCAAAAAAAATTCTGTAAAATTTTATACTTAAATTTTATATAAATGTACAGATGAAAAAGTTTTTAAAAAGATA**TCAACT**TACTGTT**G**ACAGGTGAACTCT**ATGAGAGTGTTACTAGTCGAAGATGAACCAGATTTGGGTG**

***alr0072; t*wo-component response regulator**

TTTTAAAGAAAAATGGTCTTTGAACACGATTAAAGTCTGAAAAAATTTATCAATTTTCTAGTAAAACTATCTATTGATAGATAGATAATCTGTCTCCTGGTCGAACAAATCAAAATATAAAATTGAGATAGTAATGATGATAGAAAAACACTTATCTTTATTAAAAAGCATCTAACAATCCCATCCCCAAAAATATT**ATGCGGATATTACTAGTGG**

***alr2137; two-component system, NarL family sensor kinase (Operon alr2137-38)***

TTACCAGCATGGCTGATTTAGATGCTTGGTTGTCGGGATTGGATGGTAATTCTTAAAAAGCGATACCTGCGGTAAGCTGCGCTAACGCACTTCGAGGTAGTTACTAGGTGATCGCTAACACTAAGCTGTACGCTAACGCACATTTATTCCTAACCTATTTACAGTAGTTGGTACTTTATTCGTTTGCTTGTTGGGTTTCGCTGCGTTTCACCCAACCTACAAGATATGCGATCGCACTAATTTAATTGGAGATAATAATTGTGGGGGTCTGGACGGACAGGAAAATCCCTGTGATTTTTGGTGGGGGCAGGTAAAATATTATGCAACGCTTGCGATGTCACGGGTAGGTCGTGGTTTGGATTCGGTCAAAGAGTTTCTCAGTACTCTCACTCCTGACGAGCGTTGGGGGGTGCTGGTAGTCTTTGAGGAAGTACAGCCTGTGATGTTTGACCAATTGGTGGCAATCTTTTAAAGTGAGTTAACGCTAAATCAGTAGTAACATTACGCCGTGTGCAACTTTCTCTCAAACCTAACCCCCAACCCCTTCCCTGCGAGGGAAGGGGAGCAAGATTCAAAGCCTCTCTCCGCTTCGGGGAGAGGTTTGGAGAGGGGTTTCAAAAATAAGTTGCACATCGCGTTAACATTCGTAATGTTTACGATTATGGTAATCGATCACCAGCAATTCTCATAATAAGAATTATTATCACTATCATTAGCGTCTAGATATACTCTGCCCCATGCCTTTATCCACTTTTCTTGGTCTTGCGATTGCTATTTGGGACATTCTGGGCAGAATCC**ATGCAGCCAATCTCCTCTCTGGCGCTTTTGAGCCTACCGCTCGATGCTTCAAACTCCCAAAAAGTA**

***alr2502; serine/threonine kinase, Pkn22***

ACGGGTTTTACGACGCTTGATTGTAGTATCAAAGGTGGCTTGGTTGGCTGTATTACTGGATGTTGTGGGTGTCGGCAGAGTTTGCATAACCTTAATTAGGTGGATTTGCTGTAGTTAAAATATCAGATTTAAAAGCCTATGTTTCTCAGATTGCCACAGTGGGAGCTTTTTTGAACCATGAAATGGAAAAAATCATGATCAAGAAAGGTAATTGACATTTTTATAATTCATTGTCCCACTTGAGATGAGATATTGCGATCGCCTAATTTATTACGAAGAATCCCGATCAGGT**TAAACT**ATTCTG**A**TAAAACTGTTACTTGCCGAAGCG**ATGAGCCTCTGCATAAACCCTCAATGCTCAAA**

***alr3732; protein serine-threonine kinase, PknE***

CGTATTTGGAATTTTGGCTCTAGAAAGTGAACCCGTAGATCCACGACTGTAGCGCCTATGATAAAGACAGCCAGCAGGGCTTAAACTTGACTTTAATTCATTTTTTTTGCAATGAGATCGCGATCGCTAAAATCTTTGTAA**GACAAT**AGTCTT**A**CTTTTTCAACTGCATCGGGAACCTATAAATGTGAAAAAAGTATTTGCCAAAAAGCTAGGTATTTTACCAGATAAAGAAAACCAAGTTCTTAATTTTGGATAAAGTCGAGCTTAGAGTAATTTGTCTTTCCGGAAACAGCACGCAATCAAGAG**AATACT**TATAT**A**AGGACTAACTCAGTAACCAGCTCATGAACCACCAC**ATGATCG**

***alr0709; serine/threonine kinase with two-component sensor domain***

ACATAGAAGCTAAATCAATAGCATTTGGTGAAAGGAAAGTCAGCGAAGCCTCAAAATTTATTCCTTAAGTGCTATTGACATTCGACTAAGTTTAGTTTTCCCTGATCATCTGCAAAACTCACATCAACTTAGGCTAATACCGAATGGCACTGGCTATGGTTGTGATGCCATTCTTGATGAAATTCCTTAAATCCCCGTTTTCGAGGGTTTGTACAAAAACCCCAGCTAGTGATTGAAATCAGCAGCTACTGGATTATTAGCGGCAGTTACGGCAACAACAGCAGCAACAAGACGAAAATGATTTGAGTCTTTAAATGGTTTTAAAACTGAGTCTGCATTTCTCGAAATAGCCATTTTGTATAATCCTGCATTGCTTGTAAGTGGTCACATAAGATACGCAAAAATGGTATAAATTTTTACATTTGTATAGAAGATTCAGTTAATCATTTGGGAACACTAAGTCCAAGCATTTTAAAGATTTAGGAT**ATGACTAGCACTCTTGTT**

***all0743; adenylate cyclase, CyaD***

AAAACACTAATAAAGAAAAATAGCCAATTATGTTTTTGTGGGTTTTGTTGGCTTCTCACCATAAGGAATAACTGTTAATAATTACTGGTTAATACTCAGTATCTAAATTTAGATAATGCTCATGCTTATCTAATATTATTTGCTCAAATAAAATTTTGCTTTAGCAAATGTATTTTTTCTGTAAAGATATTATGAATTAAAGATAAAGATATTTACTGATACTTAAAATATGAATTCTATTTTGGTTATTAATAAACATTGTATAACCCAAGACAAAATGAGATTAATTTATATATATACAGGTGATATTTAGAGAAGATTAAGTAATTTCTCTGAGTCACCCCTCATAGAGCTTTAAAAAGTTTTCAATATAATAGATGCGTAATAATTACTACATATAGTTGTCGATCTTCATTAAGAATGCCATAAAGAAAAGGTAAAGACTATCCTTCTATTCTTTGCCTTAGACAACTTTGTGTTCCCATCTGCCAGGATACATATATTACACCC**ATGACTGAACTGACTTTACGCTTACAACAAGGAGATACGGAAACAACTGTTACCGTGAATCGAGAT**

***al1904; adenylate cyclase, CyaB2***

GCATTGCAACCAAGTCACAAAAACTTCATCACAAGTAATGGAAAATACAACTGTTAAGTAAGGGAACACAAAAAATACATTATCCCAAAATAGCTTTTAGTAGAGTGCGTAAATATGAATAATTTCGTGGTATTTCCGGATTTTATCTGACTGACGCACCCTATATTAGGGATATTTTTTATCTGGATGTTCCGAAAAAAACTAATCCATCTGTGTACTTATCAGTAACGGTTTCCATTTTTATGCAGTAATTTCACAATTTTTTAGCAGCCGGCCGTTTCTATAAGCTTCTTACACCACCAAATATGAAAAAGGGAATAGACCGAGGGATGAGAGGTTAATATTTTTTACTGAAAAGTGTTGCCTGACCTACAAAATTGGGAACTATGATTTATACACTACCCCTATGAGTAGAGTCGGTCAAGCAAGTAATAGCTTAATATTTTGGTATAACTATTAACTTAATAATCGCCTCCGGGTTATTGTCTATACATTTTTACTGGAAGTACAGTAGTTTGTGGATGAGGACTATAAATTAGGCTAAACCAGGAATAGAGCCGGATGACGATGGCTGAACTACAGAT**TACACT**GTGTGT**A**ATGTCTATCGCTCGTTGCTTTTAGCAACACTTAAATAAATTCTTAAAAGTCTTAATCCTGTTATATCAAAAATTACCT**ATGTCATTGCAACAGCGTAATTTTGGGGAGACTGGCGATTTGATC**

***all4379; peptide-chain-release factor 3***

GGGATGGGTACGCAAACGACGAGGGCGGTGTGTTGGAAACATAAAGTTTTGTAAAAAATAACAATGGTATGGACACAAAACAAAGCGTCACAAAAGTCTGGCGAAACTCCGTTGTCGTCGGACAGACTACAAAAAGTGCTTAACTGTCCTTTGCCCTACTCTGCTTCAAGTTGTGGGGCAATTTTGTATTTTACCGTGCTGTTGCGTGTTATTAGTTGTCAGTGTTGTAGATGGGAATTTTTCCACTCGGTAGGCATCTGTATTACATCTCAAAAATCGAGAACTTTTTAAATTCTATTTGTTAATAATCAGACAATTGAGATTTGACGAAATCAGCGATCGCCTATTTTTAAATTCTCAATTTTTGGGATACATTTATGATAATTTTTATTAAAAAAGTGAGTAATGAATCTTTCTGAATGTCTGCTAACAAGGAAATAAGGCAGCAGATAGCCACCTCTTAGGGCAAACAATCACCCAGAAATCCAGCACAGGTAAACTGTATTTATCATTTCATAAAACTACGGGTTAATGAGCAACTGATTTGCTGATTTAGCTTTTTTTTAAACACCATTAAAGCTTTTTGATTTTTCTATATCTCTAGCTTGAAAAAGATCAGGGTGTAAGGGAAGGATATTTTCCTGCTTGGTTGGGAAATTTTCTACTGAGGGACTGTGTTTAAAAAGTAATTTTGTTAACCCAAGATACATAGAGGAGAATCTATTCTCAAAATCTCAGCGTATTTTGAATTTCCGGGAATAACCCTATCACGAGCATCGTTGTTTTAAGATGAAAAGACTCGCTA**AAGTAG**AAAAAAA**T**TAAGTTAAGTTACGTAACAAGGAAGTTAATACATTTT**ATGTCAACTGAAATACAGACGGAACTCCACCAAGCAGTTGAGCTTCGCCGCAATTTTGCTATTATCTCTCACCCTGACGC**

***all4963; adenylate cyclase carring two-component sensor and regulator domains, CyaC***

AAGCTTTTGCCCGTCTGCGTCCAGTAATCACTAAAACGTACGCCAACTTCTACCGTTCCCACTAACCGATCAACTCCATTGTTGGTAGTTTCCACAGCAACCAAACAAAGGTGATGGGGGGTAGGCGATAATAGGCGATGTTTGAAATCTTCATAAATACCCAAGCGGAGTAGTGGAAAAGCCCATCCCCAGAAACCATTCTGAGAGTGAAAACTTTCCGCAATAATTTGGGCGATACTAGTCAAATCAGCAGGTGTAGCAGCACGAATGTGAACCTGGTGAGAACTTGGCTTAGTATCTGCTGTGACTGGCTTGTGGCTAGATGGATCAAAAAACCAGGATGTCAAGGCTAGTTGAATATTTATATTATTCAAATAAAATCTTCTCAAATATTCTAAACAGCAACTGGGAAAATTGAACTTGTAAATTGATTTAATAGTCAATGGTCAGTGGTCAGTTGTCAACTATTATTCCATCCGTCTCCTTAGGCTTTCGCACTCTTAGCCCAATTCTGTTCGGACGGCGACGTAAAATTTTGAGACTTCGACAAAACTTAGGCAGTCTGAGCGCTCAGTGTATCACAAATTTAGTGCGAAGACTCTCAGATACTCGGCTAAGACTTCCTTGTGGGATGCTACGCTTACCTCTCTGGGGAAGCAATGCTCTGTCATATCTTTTAATACTGACGCTTACGGAGAAAGCCTAGCCCCTAATTGCTGTCCTCTTACTCTAAGATAACTGCTATAAATAAAATTTACTTAAATGTGTTAGCACAACTGTCACAACGGGCAAACTTAATATGTAGCTTCATCTAGTTGGTTTTTGCACCAGACTCGAAGAATGTATACAGTAAGACTGAAATGTTTTTGTTGGAGGTGAGGCAAGATTTGACTAATAAGCATCAAATCTGTAAAGCAAAACTTGATAACGAGACCAATTGCACTGCACTAGCGCAAATGGCAGCCCTAAAAAGCCATTGAGGCAAATTACTGTGAACCAATCCTTCCCAATGAGATTAACCTTTATCCGACTGCCGTTACAGCAGGAAAGCGGTGC**ATGAAATCCCCAGTAAACAGTAAG**
