# Supplementary material for: Regulatory networks of FUR and NtcA are intertwined by transcriptional regulators, two-component systems, serine/threonine kinases, and sigma factors in Anabaena sp. PCC 7120
Source: mSystems. 2025 Jun 25;10(7):e00373-25. doi: 10.1128/msystems.00373-25 (PMC12282138; doi:10.1128/msystems.00373-25)
Supplement: Supplemental Tables — Tables S1 and S2. [file msystems.00373-25-s0002.docx]

**SUPPLEMENTARY TABLES**

**Table S1. Oligonucleotides used in this study.**

**Table S2. Transcriptional changes of genes with regulatory functions identified as NtcA direct targets in this work at 6, 12 and 21 hours after nitrogen step-down.** Data ware obtained from Flaherty *et al.* (49).

| **Table S1. Oligonucleotides used in this study.** | | | | |
| --- | --- | --- | --- | --- |
| **EMSA** |  | |  | |
| **Primer** | **Sequence (5´→ 3´)** | **Purpose** | |  |
| Pifpkn_up | AAAGATGAATTACACTGGCG | EMSA negative control internal fragment of *pkn22* | |  |
| Pifpkn_dw | CTGCAAACTGTGGCAGAATA |  |  |  |
| **FurA regulatory network** | |  | |  |
| PsixA_up | CAAGAATTATATGCAATCGCATG | *alr0221 (sixA)* promoter | |  |
| PsixA_dw | TGCTTCAGCGATGCCATGAC |  |  |  |
| PcyaB2_up | CTGTGTACTTATCAGTAACGG | *all1904 (cyaB2)* promoter | |  |
| PcyaB2_dw | CCAAAATTACGCTGTTGCAATG |  |  |  |
| PpknE_up | AGTGAACCCGTAGATCCACG | *alr3732 (pknE)* promoter | |  |
| PpknE_dw | TTTTGAACAATTTGGTAGCGTG |  |  |  |
| PpknD_up | TCCACAGTCCACAGTCCAAG | *alr4368 (pknD)* promoter | |  |
| PpknD_dw | GAGTATCTGAATTACTTGATAGC |  |  |  |
| PrpaA_up | CCCCACAAAGTAGTGTAAACAG | *all0129 (rpaA)* promoter | |  |
| PrpaA_dw | GGCTTACATCGTAGCCAGCC |  |  |  |
| PprpA_up | CGATCGCAATTCCAAGATTG | *alr3731 (prpA)* promoter | |  |
| PprpA_dw | ATTGGAAACTAACCCACGAC |  |  |  |
| Palr0946_up | AAAGGCAAGCACTAAAAGTGG | *alr0946 (calA)* promoter | |  |
| Palr0946_dw | TTTTCCAGTTAAAGGCGCGG |  |  |  |
| Pall2080_up | CATTTTGTTAGTTGTCAATTGTC | *all2080 (calB)* promoter | |  |
| Pall2080_dw | CTGTTTAGGCATCAACTTAGG |  |  |  |
| Palr2411_up | CATAATGCACCTAACCCCTTC | *alr2411-12* promoter | |  |
| Palr2411_dw | GTCCTAATTGACGTATGATGCG |  |  |  |
| Pall4896_up | TTATCAGTACAATGGCAGATTG | all4896 promoter | |  |
| Pall4896_dw | TTTCATTAGTCACTTAAGATAGC |  |  |  |
| Pall1651_up | GAAACCCGTCCAACGCAGTG | *all1651* promoter | |  |
| Pall1651_dw | GCTGCATATAGTGTTAATTTAGG |  |  |  |
| Pall2416_up | TAGTTATCTATACTTAATTCCTCG | *all2416* promoter | |  |
| Pall2416_dw | GAATTGTCATAGTCATGGCTG |  |  |  |
| Pall1804_up | GAGATTAGCTAGTATTGCTCC | *all1804* promoter | |  |
| Pall1804_dw | AAATAGCCTCTTAGATGGTTAC |  |  |  |
| Pall4379_up | CTACTCTGCTTCAAGTTGTGG | *all4379* promoter | |  |
| Pall4379_dw | GCTAAATCAGCAAATCAGTTGC |  |  |  |
| Pall3903_up | CAAGTCTGCAATCAAATAAGTG | *all3903* promoter | |  |
| Pall3903_dw | CAGATTTAAGCGCACACAATAC |  |  |  |
| Palr0072_up | GACGTAAATCAGGCATAGACG | *alr0072* promoter | |  |
| Palr0072_dw | AGCCAGCTTCCGATAAGATAC |  |  |  |
| Pall1704_up | CAAAATACCCTACGGGAAGC | *all1704* promoter | |  |
| Pall1704_dw | GCGCAATCCGAATTTTATTCAT |  |  |  |
| Pall7584-1_dw | TATAGCGTCAAACTCGAATATG | *all7584-83* promoter  (proximal region) | |  |
| Pall7584-1_dw | CTTCGACTAGTAACACTCTCA |  |  |  |
| Pall7584-2_up | GTGCTATGGCAATTGGTGTG | *all7584-83* promoter  (distal region) | |  |
| Pall7584-2_dw | CACATCAAGAAAGTTCATTAGC |  |  |  |

| **FurB regulatory network** | |  |
| --- | --- | --- |
| Pall3767_up | CACACCAACGCTCGTTG | *all3767-64* promoter |
| Pall3767_dw | CATCTTATGACGAAGTATTGTC |  |
| Pall3768_up | CTTACTTTCATAAAGTTGAC | *all3768 (orrA)* promoter |
| Pall3768_dw | CACTCATCGTTACACTCACTC |  |
| Pall0926_up | CGCACTGCAAAACCTAGTGG | *all0926* promoter |
| Pall0926_dw | ACTTTCAAGATTGAGCAACCC |  |
| Pall4986_up | TACTCATAGGATCAATAGCGG | *all4986* promoter |
| Pall4986_dw | CTCAAATGCTCTAAGCTTAAAG |  |
| Pall5210_up | CTGCATAAGTGTTAACAGTTAC | *all5210* promoter |
| Pall5210_dw | CAGAAATAGGAATATTAGGCATG |  |
| Pall7606_up | GCTAGAGTTTGCCGAATCAG | *all7606* promoter |
| Pall7606_dw | CAAAGATTGCCAGTGTATATTG |  |
| Palr0428_up | CCATATTAGATTCCTGAGTGAG | *alr0428* promoter |
| Palr0428_dw | ATTGTTGATCAGGTAATGGGC |  |
| Palr0900_up | AGAAGCATCAGCCGCTATAC | *alr0900* promoter |
| Palr0900_dw | TCCAGACAGCAAAACGGTTG |  |
| Palr7219_up | GATTTGCAATCAACGATATTCC | *alr7219* promoter |
| Palr7219_dw | TCGGATTAATTCCTCATCCTC |  |
| Palr8535_up | AAATGATTGGCACTCTCATG | *alr8535* promoter |
| Palr8535_dw | GTAGCACGTAGACCAATTCG |  |
| **FurC regulatory network** | |  |
| Pall0345_up | CTCACTTAACAGTCAATAATC | *all0345* promoter |
| Pall0345_dw | CAACTGTCTACAGTACACAC |  |
| Pall2035_up | GGAAGCTGCACAACATCTTTG | *all2035* promoter |
| Pall2035_dw | GACACAATCCATCAGCAAATTA |  |
| Palr1941_up | TGCTTGGTTGGAGAGCAATTG | *alr1941* promoter |
| Palr1941_dw | GTGCGTGTCTGTGCCTCTG |  |
| Palr1976_up | ACGCGGATATGGTACAGCAG | *alr1976* promoter |
| Palr1976_dw | ACTAGGCTCGCCAAGCGTTC |  |
| Pall3564_up | CAACACCTTTAGAAATGCGAG | *all3564* promoter |
| Pall3564_dw | GAATCGTACTTTCTTCTAGTC |  |
| Pall1071_up | GGATCTCGTTTAGAATTCTGTC | *all1071-68* promoter |
| Pall1071_dw | AATTTCGCGGTCGGTGAGAC |  |
| Pall1281_up | CAGATTGTGAGGCAGTCATAG | *all1281* promoter |
| Pall1281_dw | CTGACAACGGTAGTGTCATCG |  |
| Pall3359_up | CTTGCAAGCCCAATCTGCCA | *all3359* promoter |
| Pall3359_dw | CGCTACTTCTACACCATTCAG |  |
| Pall3788_up | GCACGTAAGCAGAAAGACATAC | *all3788* promoter |
| Pall3788_dw | GATATATGAGGTTACGGACTCC |  |
| Pall4687_up | CAGTATGAGTTCTATATGATCTC | *all4687* promoter |
| Pall4687_dw | GTTGTTCTGTAATGCGATATCC |  |
| Pall5323_up | GGAGAGACAAGCAAGACTCG | *all5323* promoter |
| Pall5323_dw | GCCATAGCTTGAGCCAGCAG |  |
| Palr0264_up | TAGGCAAAGGTAACTTTAGTTG | *alr0264* promoter |
| Palr0264_dw | GCTGCTGAGAGTAGTGCTTC |  |

| Palr0354_up | | GTGTTTGTGAGGGAATTTAATC | | *alr0354* promoter | |
| --- | --- | --- | --- | --- | --- |
| Palr0354_dw | | GAACCATTATAAAGTTCTTCGC | |  |  |
| Palr0709_up | | GTTCCTGAAAATTGACATAGAAG | | *alr0709* promoter | |
| Palr0709_dw | | TTCTCAACAAGAGTGCTAGTC | |  |  |
| Palr0774_up | | GAATGTGGAGAAGATATCACAG | | *alr0774* promoter | |
| Palr0774_dw | | CAGAAATCGTGCTAGTTTGAC | |  |  |
| **Sigma factors** | |  | |  | |
| Pall5263-1_up | | GAGCCTTGACCCTACTTAATCAG | | *all5263 (sigA)* promoter  (proximal region) | |
| Pall5263-1_dw | | GCCTGGTTCATGCCGCGTTC | |  |  |
| Pall5263-2_up | | CTACGAGCTAGCGGTGATGAAC | | *all5263 (sigA)* promoter  (middle region) | |
| Pall5263-2_dw | | CCGATTGTAGCCTGTTTCACAG | |  |  |
| Pall5263-3_up | | CGTGAGCCTGACTATGCTTAAG | | *all5263 (sigA)* promoter  (distal region) | |
| Pall5263-3_dw | | CGTGTTCATCACCGCTAGC | |  |  |
| Pall5263-4_up | | GGGGCAGAGGTGGCAGAG | | *all5263 (sigA)* promoter  (distal-far region) | |
| Pall5263-4_dw | | GTCAGGCTCACGTTACAAACAAG | |  |  |
| Pall7615_up | | CACCTGTCGCCAATTAGGAC | | *all7615 (sigB)* promoter | |
| Pall7615_dw | | CTCTTAAGTTTAGTCACTTGGGATG | |  |  |
| Pall1692_up | | CCCATGTAGATGTGACTATGC | | *all1692 (sigC)* promoter | |
| Pall1692_dw | | GTTGCTGGCATAAGATCGTC | |  |  |
| Palr3810-1_up | | CTACTCAGTCCCTGAACAATCTGC | | *alr3810 (sigD)* promoter  (proximal region) | |
| Palr3810-1_dw | | GGTAGGGTCATTAACTGAATAGATG | |  |  |
| Palr3810-2_up | | CCTCTCCCCCCACTCCTAC | | *alr3810 (sigD)* promoter (distal region) | |
| Palr3810-2_dw | | CAGGGACTGAGTAGAATCGTTTAAC | |  |  |
| Palr4249-1_up | | GATTGCAGTTATGTCTCTCAACG | | *alr4249 (sigE)* promoter  (proximal region) | |
| Palr4249-1_dw | | CCTTGAGGGATTCATGCTTTG | |  |  |
| Palr4249-2_up | | GCCTTAAACTCAGTCCGTAAAGTTC | | *alr4249 (sigE)* promoter  (distal region) | |
| Palr4249-2_dw | | CGTTGAGAGACATAACTGCAATC | |  |  |
| Pall3853_1_up | | AAAGCTCCAAGAAAATAGCCG | | *all3853 (sigF)* promoter  (proximal region) | |
| Pall3853_1_dw | | CAACTGCCAAATTTCATACTTC | |  |  |
| Pall3853_2_up | | GACAGGGAGTTGGGAAAAATG | | *all3853 (sigF)* promoter  (middle region) | |
| Pall3853_2_dw | | GAGTAACACAAAGACCAAAACC | |  |  |
| Pall3853_3_up | | CTCAACGGCAAAATTTCTCTAC | | *all3853 (sigF)* promoter  (distal region) | |
| Pall3853_3_dw | | CTTTTTACCACTGATTGAACTG | |  |  |
| Palr3280_1_up | | TTCTATGAAAACCAGTTGCAGAG | | *alr3280 (sigG)* promoter  (proximal region) | |
| Palr3280_1_dw | | AGAGTTTGTCAACTTGCACCGAT | |  |  |
| Palr3280_2_up | | CCTGGTATCCGACTTAAAAG | | *alr3280 (sigG)* promoter  (distal region) | |
| Palr3280_2_dw | | CAATACGCAGATTGTTATTTAC | |  |  |
| Palr0277_up | | TTAGCCTAACGGGATGCAAG | *alr0277 (sigJ)* promoter |  |  |
| Palr0277_dw | | TACCATCAGTTTGCATAGAGG |  |  |  |
| Pall2193_up | | TTAGATGAAGATGTTGCTGAAG | *all2193 (sigI)* promoter |  |  |
| Pall2193_dw | | AGCTTCATCCGTCACATCGG |  |  |  |
| Palr3800_up | | CGGTTATCTCCCTACTGGCAG | *alr3800 (sigB2)* promoter |  |  |
| Palr3800_dw | | GCATTGGTGCGTTGTCCTC |  |  |  |
| Pall7608_up | | GAGCTTTATTACTCGCTCTATC | *all7608 (sigB3)* promoter |  |  |
| Pall7608_dw | | CAATATCTTGCAGATAATTCCG |  |  |  |
| Pall7179_up | | GTGAAATACCTTGCTTGAGC | *all7179 (sigB4)* promoter |  |  |
| Pall7179_dw | | GGTCAGAGGTTAACAAAGGG |  |  |  |
| **NtcA corregulation** | |  |  |  |  |
| Pall2416_ntcA_up | | CACTTGATATTGAGCTTAAGTG | *all2416* promoter  (NtcA box) |  |  |
| Pall2416_ntcA_dw | | ATTATAGCCACACAATCACTG |  |  |  |
| Pall1804_ntcA_up | | CATAGAAGCACTTTACCAGG | *all1804* promoter  (NtcA box) |  |  |
| Pall1804_ntcA_dw | | GAGATTAGTCGAACGCTTG |  |  |  |
| Pall4379_ntcA_up | | GTTAACCCAAGATACATAGAG | *all4379* promoter  (NtcA box) |  |  |
| Pall4379_ntcA_dw | | CCAGCTTCGTGAATTGCAC |  |  |  |
| Pall7584_ntcA_up | | AGACTCGATATTTCTATCCTG | *all7584* promoter  (NtcA box) |  |  |
| Pall7584_ntcA_dw | | TGAGGAGTTACAGAAACTAAG |  |  |  |
| Pall2080_ntcA_up | | CTATTAGCTCAACGTCAGC | *all2080 (calB)* promoter  (NtcA box) |  |  |
| Pall2080_ntcA_dw | | GTTACCATTCGATTGCACAC |  |  |  |
| PpknE_ntcA_up | | TTTCAACTGCATCGGGAACC | *alr3732 (pknE)* promoter  (NtcA box) |  |  |
| PpknE_ntcA_dw | | GGTGACTAGAGTGGAAACTG |  |  |  |
| PrpaA_ntcA_up | | TCCCTACAATCGTACTGTGG | *all0129 (rpaA)* promoter  (NtcA box) |  |  |
| PrpaA_ntcA_dw | | AGTTCTGAAATCGCTGGGTC |  |  |  |
| PcyaB2_ntcA_up | | GGTCGATCATCACCATCATG | *all1904 (cyaB2)* promoter  (NtcA box) |  |  |
| PcyaB2_ntcA_dw | | CCGTTACTGATAAGTACACAG |  |  |  |
| Palr0428_ntcA_up | | GTAAATAGGTGAAAGTAGGAG | *alr0428* promoter  (NtcA box) |  |  |
| Palr0428_ntcA_dw | | CTAAATCGCCTCTCTTGGTC |  |  |  |
| Palr7219_ntcA_up | | CCCGGTCATTAGTATCTCG | *alr7219* promoter  (NtcA box) |  |  |
| Palr7219_ntcA_dw | | TCGGATTAATTCCTCATCCTC |  |  |  |
| Palr2137_ntcA_up | | CCTTCCCTTAGAACAATTGG | *alr2137* promoter  (NtcA box) |  |  |
| Palr2137_ntcA_dw | | GTGACATCGCAAGCGTTG |  |  |  |
| Palr0264_ntcA_up | | GCTATGGCATATTAGCTACC | *alr0264* promoter  (NtcA box) |  |  |
| Palr0264_ntcA_dw | | CGTTGAGACCGATAACTCGC |  |  |  |
| Palr0709_ntcA_up | | CCGTTTTCGAGGGTTTGTAC | *alr0709* promoter  (NtcA box) |  |  |
| Palr0709_ntcA_dw | | TAAACTAGTGTACGGGAGCC |  |  |  |

| **RT-PCR** |  |  |
| --- | --- | --- |
| **Primer** | **Sequence (5´→ 3´)** | **Purpose** |
| RT_all5263_fw | TGGGCAGAAGCCGTACAATT | *all5263 (sigA)* |
| RT_all5263_rv | GCTCTGCGGCCAATGTG |  |
| RT_all7615_fw | TCTCAAAAGCTTGGTTACATTGCT | *all7615 (sigB)* |
| RT_all7615_rv | TCGAATCTGACTAGGTTCCAAATTG |  |
| RT_all1692_fw | TGATTTGTCGGTGGATGAGATC | *all1692 (sigC)* |
| RT_all1692_rv | ACGACGGTTAGCGCTAGCA |  |
| RT_alr3810_fw | CACCGAGAAGCTGAACAAAATTAA | *alr3810 (sigD)* |
| RT_alr3810_rv | GGCGCACGTCCCAATCT |  |
| RT_alr4249_fw | GGAGGAAGTCGGAAATATGTTCAA | *alr4249 (sigE)* |
| RT_alr4249_rv | TTACGCATGGCTTTGCTTTG |  |
| RT_all3853_fw | TTGCCAAAGGCCATGCTT | *all3853 (sigF)* |
| RT_all3853_rv | TCGCGGAGGTAGTGTTGAATT |  |
| RT_alr3280_fw | ACGCAAGCGGGTTGTAAGTC | *alr3280 (sigG)* |
| RT_alr3280_rv | CAATCCATTTCGCCATCATCT |  |
| RT_alr0277_fw | TTAGGTGAAGCCCTTCCTTGTC | *alr0277 (sigJ)* |
| RT_alr0277_rv | TTGCAGTTGTTGCCGTTCTT |  |
| RT_all2193_fw | GGCTTTGAAAATTCTGGCTGAT | *all2193 (sigI)* |
| RT_all2193_rv | TGCGCCACAGTGTGAGAAA |  |
| RT_alr3800_fw | CGATTGAACCTCAGTCGTGAAC | *alr3800 (sigB2)* |
| RT_alr3800_rv | CGACGGCGACGCAGAT |  |
| RT_all7608_fw | TTCTACCTACGCTTATTGGTGGATT | *all7608 (sigB3)* |
| RT_all7608_rv | TCGGTAATATGAATTGGCAGTCTAAT |  |
| RT_all7179_fw | GTTGCGCCAAGACTTAAGTGATT | *all7179 (sigB4)* |
| RT_all7179_rv | CCAACAGTCCAAAACGTAGGATT |  |
| RT_alr2137_fw | GCGCGAGGTCGGTCAA | *alr2137-38* |
| RT_alr2137_rv | CGCCCCAGCGATCGT |  |
| RT_alr2325_fw | GGCGCACCTGTGGAGATG | *alr2325 (ancrpB)* |
| RT_alr2325_rv | CCAAAAAAATCACCCCTTCCTA |  |
| RT_calB_fw | TGCGGAAGGCATTCAGTTAGA | *all2080 (calB)* |
| RT_calB_rv | GAACCAATCAATAAGTTACCATTCGA |  |
| RT_rpaA_fw | TGATGTTGCCTAGAGTTGATGGTT | *all0129 (rpaA)* |
| RT_rpaA_rv | TTTCTGCCGTCCGTTCGT |  |
| RT_all1804_fw | TGCTTTTGGGTGATGTGCTTAC | *all1804* |
| RT_all1804_rv | TGCCTATGAGGAGAAAATTAATTGC |  |
| RT_all7584_fw | AGATTGCGGGCATTGCA | *all7584-83* |
| RT_all7584_rv | CGTAATCTAGAGTTAAGTTGCCAACAGT |  |
| RT_cyaC_fw | CGGTTGTGGGAATGTTTGGT | *all4963 (cyaC)* |
| RT_cyaC_rv | AATCGAGCAGCGATATTGACACT |  |
| RT_cyaD_fw | CGGTGATTGTATCATGGCATTT | *all0743 (cyaD)* |
| RT_cyaD_rv | GATCGGCGTGGTCTGCTT |  |
| RT_cyaB2_fw | TGACCCAACTGGTAAATAAAAAGAAA | *all1904 (cyaB2)* |
| RT_cyaB2_rv | GGCGCTATCGGCCAAGT |  |
| RT_all4379_fw | GCGATCGCTTCAAGGGAGTA | *all4379* |
| RT_4379_rv | CCGTGAGCGCTACGTTCAA |  |
| RT_pkn22_fw | GCCTTGGGACGTACTTTCATTT | *alr2502 (pkn22)* |
| RT_pkn22_rv | CACGCCAGTGTAATTCATCTTTTAA |  |
| RT_alr0709_fw | GGCAGAATGAATCGAGGGATT | *alr0709* |
| RT_alr0709_rv | GGTAACCCACCTGTAAGTAATTCGTAGA |  |
| RT_pknE_fw | GCTGATACGCCTGTGAGTGACT | *pknE (alr3732)* |
| RT_pknE_rv | TGAAAGCGTTCTTGGAAATTGTAG |  |
| RT_rpnB_fw | AGCGGAACTGGTAAAAGACCA | *rnpB housekeeping* |
| RT_rpnB_rv | GAGAGGTACTGGCTCGGTAAAC |  |

| **Table S2. Transcriptional changes of genes with regulatory functions identified as NtcA direct targets in this work at 6, 12 and 21 hours after nitrogen step-down.** Data ware obtained from Flaherty *et al.* (49). | | | | | | |
| --- | --- | --- | --- | --- | --- | --- |
|  | **Putative transcriptional unit^1^** | **Gene name / protein description** | | **Fold change under nitrogen deprivation** | | |
|  |  |  |  | **6 h** | **12 h** | **21 h** |
| ***Transcriptional regulators*** | | |  |  |  |  |
|  | *all2080* | ***calB***; AbrB family transcriptional regulator | |  |  | **3.3** |
| ***Two component systems*** | | |  |  |  |  |
|  | *all0129* | ***rpaA***; two-component response regulator | |  |  | **2.2** |
|  | *all1804* | *all1804*; two-component hybrid sensor and regulator | |  |  |  |
|  | *all7584-83* | *all7583*; two-component sensor histidine kinase | |  |  |  |
|  |  | *all7584*; two-component response regulator | |  |  |  |
|  | *alr2137-38* | *alr2137*; two-component system, NarL family, sensor kinase | |  | **3.2** | **2.0** |
|  |  | *alr2138*; two-component response regulator | | **4.3** | **9.7** | **2.4** |
| ***Serine/Threonine kinases*** | | |  |  |  |  |
|  | *alr2502* | ***pkn22***; serine/threonine kinase | |  | **2.2** |  |
|  | *alr0709* | *alr0709*; serine/threonine kinase with two-component sensor domain | |  |  | **3.1** |
|  | *alr3732* | ***pknE***; protein serine-threonine kinase | |  | **3.8** |  |
| ***Other genes with regulatory functions*** | | |  |  |  |  |
|  | *all0743* | ***cyaD***; adenylate cyclase | |  |  |  |
|  | *all1904* | ***cyaB2***; adenylate cyclase | | **2.3** | **5.2** | **4.9** |
|  | *all4379* | *all4379*; peptide-chain-release factor 3 | |  | **2.2** | **2.1** |
|  | *all4963* | ***cyaC***; adenylate cyclase carring two-component sensor and regulator domains | |  | **2.7** |  |
| ***Sigma Factors*** | | |  |  |  |  |
|  | *all5263* | *sigA* | |  | **2.0** | **2.1** |
|  | *all7615* | *sigB* | |  | **2.1** | **4.7** |
|  | *all1692* | *sigC* | |  | **11.7** | **5.0** |
|  | *all7608* | *sigB3* | |  |  |  |
|  | *all7179* | *sigB4* | |  |  |  |
|  | *alr3810* | *sigD* | |  |  |  |
|  | *alr4249* | *sigE* | |  | **2.3** | **2.7** |
|  | *alr3280* | *sigG* | |  |  |  |
|  | *all2193* | *sigI* | |  |  |  |
|  |  |  | |  |  |  |
|  |  |  | |  |  |  |
|  |  |  | |  |  |  |
|  |  |  | |  |  |  |
|  |  |  | |  |  |  |
